# *De novo* transcriptome provides insights into the growth behaviour and resveratrol and trans-stilbenes biosynthesis in *Dactylorhiza hatagirea* - An endangered alpine terrestrial orchid of western Himalaya

**DOI:** 10.1038/s41598-019-49446-w

**Published:** 2019-09-11

**Authors:** Nisha Dhiman, Nitesh Kumar Sharma, Pooja Thapa, Isha Sharma, Mohit Kumar Swarnkar, Amit Chawla, Ravi Shankar, Amita Bhattacharya

**Affiliations:** 1 Division of Biotechnology, CSIR-Institute of Himalayan Bioresource Technology, Palampur, 176061 H.P. India; 2 Academy of Scientific and Innovative Research(AcSIR), CSIR-Institute of Himalayan Bio-Resource Technology, Palampur, 176061 Himachal Pradesh India

**Keywords:** Computational biology and bioinformatics, Transcriptomics

## Abstract

This is the first report on *de novo* transcriptome of *Dactylorhiza hatagirea*, a critically-endangered, terrestrial orchid of alpine Himalayas. The plant is acclaimed for medicinal properties but little is known about its secondary-metabolites profile or cues regulating their biosynthesis. *De novo* transcriptome analysis was therefore, undertaken to gain basic understanding on these aspects, while circumventing the acute limitation of plant material availability. 65,384 transcripts and finally, 37,371 unigenes were assembled *de novo* from a total of 236 million reads obtained from shoot, tuber and leaves of the plant. Dominance of differentially-expressing-genes (DEGs) related to cold-stress-response and plant-hormone-signal-transduction; and those involved in photosynthesis, sugar-metabolism and secondary-metabolite-synthesis provided insights into carbohydrate-partitioning in the plant during its preparation for freezing winter at natural habitat. DEGs of glucomannan, ascorbic acid, carotenoids, phylloquinone/naphthoquinones, indole alkaloids, resveratrol and stilbene biosynthesis revealed the secondary-metabolite profile of *D. hatagirea*. UHPLC results confirmed appreciable amounts of resveratrol and trans-stilbene in *D. hatagirea* tubers, for the first time. Expression analysis of 15 selected genes including those of phenylpropanoid pathway confirmed the validity of RNA-seq data. Opportunistic growth, temperature- and tissue-specific-differential-expression of secondary metabolite biosynthesis and stress tolerant genes were confirmed using clonal plants growing at 8, 15 and 25 °C.

## Introduction

*Dactylorhiza hatagirea* (D.Don) Soo (family Orchidaceae) is a critically endangered terrestrial orchid having palm shaped tubers. The vernacular name of the plant is ‘salam-panja’ meaning ‘the palm of a hand’. The plant is native to alpine and sub-alpine Himalayas (2,800 to 4,000 m amsl) but may also grow along grassland slopes of temperate climatic zones^1^. The orchid is known for a range of medicinal properties^2,3^. Its below-ground parts (tubers) are used for treating chronic fever, cold, cough, pyorrhea, cuts, wounds and intestinal disorders. It is also used for treating bone fractures, urinary tract infections, seminal weakness, diabetes and sexual dysfunctions like low sperm count, spermattorrhoea, lack of vigour etc.^4–6^. The roots of the plant are used in the preparation of nervine tonics because of their emollient and rejuvenating properties. The plant is also used in the treatment of hemiplegia, cerebropathy, phthisis and neurosthania^7^.

The tubers of *D. hatagirea* yield ‘Salep’, a high quality astringent having aphrodisiac properties. Salep is extensively used in the Ayurveda, Siddha, Unani, Amchi and other alternative systems of medicine^3,8^. Salep obtained from many species of orchids is popularly used in hot beverages, farinaceous foods, ice-creams and medicines^9,10^. Although *D. hatagirea* is expected to have a rich reserve of several high value phytochemicals, only compounds like dactylorhin A to E, dactylose A and B, and Salep have been reported till date. While the annual demand for the plant is about 5,000 tonnes^4^, the consumption of Salep is about 7.38 tonnes^11^. This drives a flourishing trade of about US $ 71,583. The dried rhizomes alone sell at US $ 72 per kg and each mg of dactylorhin E is priced at about US $ 311.49 (Sigma-Aldrich, Merck KGaA, Darmstadt, Germany).

Despite these advantages, information regarding the complete secondary metabolite profile of the plant, and the molecular cues regulating their biosynthetic routes for survival in alpine Himalayas is lacking. Resolution of these issues can be quite challenging in endangered Himalayan plants from inaccessible locales. This is because of the extremely low availability of plant material for carrying out extensive studies. The Next Generation Sequencing approach on the other hand, uses only a limited amount of tissues for a rapid, accurate and effective study on the biology of plants that have not been studied so far^12^. The approach has proven particularly useful in the identification of totally new/novel genes and key regulators of various metabolic and phenological processes operative in plants and other life-forms inhabiting habitat extremes^13–15^. Hence, the approach was successfully used to understand the evolution and adaptation of two Euro-Siberian species of *Dactylorhiza* i.e., *D. incarnata* and *D. fuchsii*^16^. However, there are no reports available on gene expression studies in *D. hatagirea* using either RNA sequencing or other approaches.

Hence, the present study aimed at gaining insights into (i) discovery transcriptome of *in situ* plants of *D. hatagirea*, (ii) comparative transcriptomes of different plant parts, (iii) response of the plant to declining air temperatures during September, i.e., the month before the onset of freezing winter and (iv) secondary metabolite synthesis. The present study being the first report on the *de novo* transcriptome of *D. hatagirea*, was envisaged to open up avenues for genetic modulation of secondary metabolite(s) biosynthesis and effective conservation of this critically endangered alpine orchid.

## Results

### Documentation of seasonal and vegetation characteristics at natural habitat of *D. hatagirea*

May constitutes ‘spring’ at the habitat niche of *D. hatagirea* (i.e., region below Sach Pass; 3360 m amsl; latitude: 74°47′24″ and longitude: 34°5′24″N). Spring at the region is characterized by snow-melt, an average day temperature of approximately, 15 °C and new growth (bud sprouting) in perennating herbs. June to mid-July comprises of summer months, when the average day temperature ranges between 25 to 30 °C and most herbs show luxuriant growth. The rainy season is comprised of mid-July to August. Thereafter, during September, the average day temperature declines to 15 °C and aerial parts of several herbaceous species become senescent (yellow leaves). The month signifies the end of growth season, and also the period before winter. Mid-October marks the beginning of winter. Based on these observations, September was presumed to be the period when plants initiated their cold adaptive strategies and made preparations for freezing stress.

### Transcriptome sequencing and *de novo* assembly

The Illumina short-read sequencing and assembly were undertaken for the *de novo* transcriptomes of *D. hatagirea* plant parts. The aim was to propose how the plant partitioned carbohydrates and used it for the synthesis of secondary metabolites as preparation for oncoming stress of freezing winter at natural habitat. A total of 6 cDNA libraries prepared from the total RNA of leaves, shoots and tubers (2 replicates each because the plant material was not enough to have more replicates) were subjected to paired end sequencing (2 × 72 bp) on Illumina GA IIx platform. Raw reads (fastq format) were submitted to NCBI (Accession no. PRJNA531300). After the initial pre-processing, an average of 72.67, 68.71 and 72.63% reads were obtained for each plant part (Fig. S1; Supplementary Table S1). D*e-novo* assembly of these high-quality reads was performed using the SOAPdenovo-Trans tool at varying K-mer length i.e., 21 to 67 base pairs (bp). Best performing assembly was selected using parameters like maximum length of transcripts, average transcript length, percentage of transcripts having length more than 1,000 bp and N50 value. A total of 65,384 transcripts having a maximum length of 10,934 bp, 17.786% transcripts above 1000 bp, N50 value of 1,057 bp and average contig length of 551.10 bp were the best performing assembly at 49-kmer (Supplementary Table S2). Further improvement by clustering approaches like TGICL-CAP3 and CD-HIT yielded a total of 52,581 and 43,873 sequences, respectively. Out of 43,873 transcripts from CD-HIT clustering, 30,197 found blast hits in nr database (weblink-ftp://ftp.ncbi.nlm.nih.gov/blast/db/). Based on dissimilar sequence (DS) clustering approach, the transcripts were merged further (Supplementary Table S3). This resulted in 21,695 transcripts. Transcripts (13,676) which did not have any significant hit in nr database were investigated for conserved domain in CDD (Conserved Domain Database) using RPS-blast. After DS clustering approach, the transcript number reduced from 65,384 to 35,371. The maximum length was 10,934 irrespective of approach, yet, the average base pair length on transcripts improved from 551.10 bp to 650.08 bp and percentage of transcripts above 1000 bp increased from 17.79 to 19.61. A total of 245 unigenes returned significant hits in CDD database. Out of these 245 domains, a maximum of eight unigenes belonged to RNA recognition motif (RRM) superfamily domain, hinting at their possible RNA binding roles (Fig. S2).

### Functional annotation and classification

Annotation of all transcripts against known functional proteins was attempted. Primarily 43,873 transcripts from CD-HIT were searched against nr database for clustering purpose. Transcripts having significant hits in nr database were classified as per gene ontology terms. In total, 21,695 unigenes from DS-clustering were used and 16,941 out of 21,695 transcripts were annotated in at least one of the ontology category i.e., biological processes, cellular components or molecular functions. In the biological process category, maximum number of genes belonged to response to cold (1.22%) followed by salt stress (1.02%) (Fig. S3a). In molecular function category, protein binding (19.50%), sequence specific DNA binding transcription factor activity (3.01%), RNA binding (2.81%), mRNA binding (2.12%) and ubiquitin-protein ligase activity (1.61%) were the major classes (Fig. S3b). In case of cellular components, the predominant categories were nucleus (15.76%), cytosol (7.06%), plasma membrane (6.82%), chloroplast (5.61%) and mitochondria (5.21%) (Fig. S3c). The dominant categories in GO analysis included the up-regulated expression of cold related genes (biological process), nucleus and chloroplast (cellular component) and protein binding (molecular function). Top 20 up and down regulated GO enriched categories were recorded (Fig. S3a i-iii, b i-iii, c i-iii). Maximum number of genes belonged to cold stress response, irrespective of plant parts in biological process category.

In an attempt to elucidate various biochemical pathways operative in *D. hatagirea*, total of 9,130 transcripts were assigned to various KEGG pathways. The pooled data set for KEGG pathways revealed plant hormone signal transduction (474 out of 9,130 transcripts) as the most abundant pathway (Fig. S4a). Comparison of pathways in plant parts revealed plant hormone signal transduction and starch and sucrose metabolism as the most abundant pathways in ‘shoot versus tubers’. In ‘shoot versus leaf’ and ‘tuber versus leaf’, plant hormone-signal-transduction was the third most abundant pathway after photosynthesis and ribosome (Fig. S4a i, ii and iii). Sequence search against enzyme classification categories revealed non-specific serine/threonine protein kinase, RING-type E3 ubiquitin transferase, RNA helicase, peptidyl prolyl isomerase and RNA-directed DNA polymerase as most abundant classes. The representative top 20 categories in enzyme classification are depicted in Fig. S5.

Similarity search of sequences against different species revealed maximum homology with the orchids, *Dendrobium catenatum* and *Phalaenopsis equestris*. The other species were *Elaeis guineesis*, *Asparagus officinalis* and *Ananas comosus*. The top 20 species showing homology with *D. hatagirea* in blast similarity search are shown in Fig. S6.

### Tissue specific DEGs

**Figure 1:**
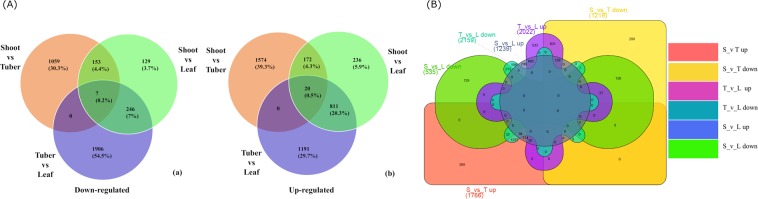
(**A**) Venn diagram showing number of common and specific DEG’s in pair wise comparison among different plant parts of *Dactylorhiza hatagirea*; (a) down-regulated (b) up-regulated. (**B**) Venn diagram showing six categories of specific number of DEG’s in tissue specific comparison of plant parts i.e., tuber (T), shoot (S)and leaf (L) of *Dactylorhiza hatagirea*; Category 1: up-regulated genes in S v T up, Category 2: down regulated genes in S v T down, Category 3: up-regulated genes in T v L up, Category 4: down regulated genes in T v L down, Category 5: up-regulated genes in S v L up, Category 6: down regulated genes in S v L down.

**Figure 2:**
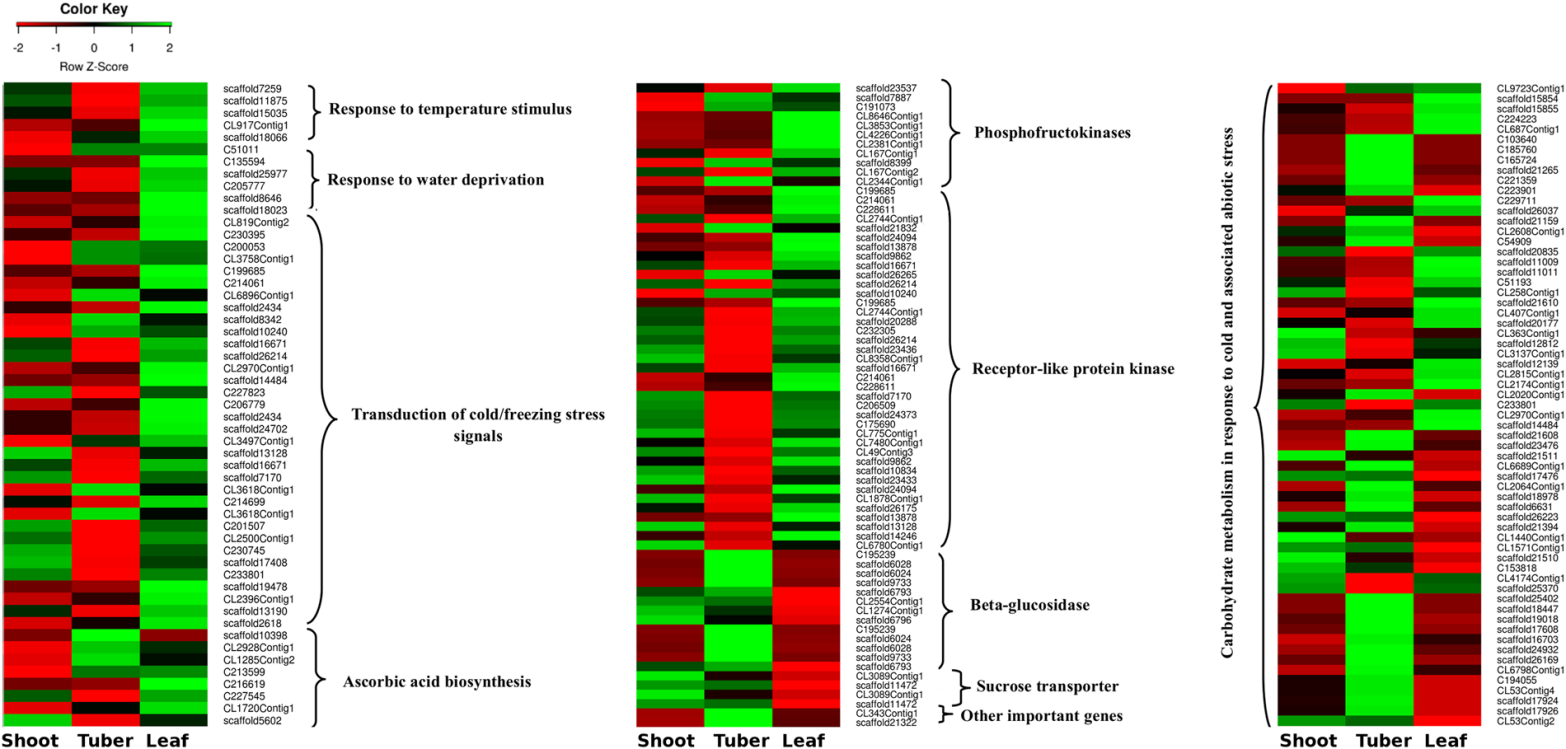
Heatmaps of selected differential expressing unigenes involved in various steps of plant response to cold stress in *D. hatagirea*.

The edgeR software was used and transcripts having p-value below 0.05 and log fold change >2 were considered as DEGs. Pair wise comparison of transcripts in plant parts resulted in a total of 2985 DEGs (1219 down, 1766 up-regulated) in shoot versus tuber, 1774 DEGs (535 down and 1239 up-regulated) in shoot versus leaf and 4181 DEGs (2159 down and 2022 up-regulated) in tuber versus leaf (Fig. 1A, Fig. S7). DEGs above log fold change >4 are presented in Supplementary Table S4. While the unigenes specific to each tissue are depicted in Fig. 1B, the DEGs involved in various steps of plant response to cold stress are depicted in Fig. 2 and Supplementary Table S5.

### Integration of GO enrichment and KEGG pathway

GO enrichment analysis of DEG’s of different tissues, having log fold change value of >2 revealed corresponding matches of enriched GO categories and KEGG pathways in leaves, shoot and tubers (Supplementary Table S6). Thus, in leaves compared to shoots, transcription factor PIF4-like, zeaxanthin-7,8(7′,8′)-cleavage-dioxygenase-chromoplastic-isoform-X2, glyceraldehyde-3-phosphate-dehydrogenases, glutamate/glyoxylate-amino-transferase-2-like etc. of the GO enriched process, abiotic stress response (GO id 0009628) integrated well with enriched KEGG pathways like circadian rhythm of plants, carotenoid biosynthesis and carbon fixation in photosynthetic organisms. The prolycopene isomerase of the GO enriched process, plastid organization (GO id: 0009657) matched with carotenoid biosynthesis of KEGG pathway, whereas, the 2-carboxy-1,4-naphthoquinone phytyltransferase of GO enriched process with GO id, 0006091 integrated with the ubiquinone and other terpenoid-quinone biosynthesis of enriched KEGG pathway. The photosynthesis antenna proteins of KEGG pathway also matched with chlorophyll a/b binding proteins CP29.1, CP29.2, CP26 and LHCII type I of GO enriched processes like response to radiation (GO id: 0009314), light (GO id: 0009416) and blue light (GO id: 0009637). MAP kinase signalling of KEGG pathway, correlated well with the protein phosphatases of GO enriched processes *viz*., generation of precursor metabolites and energy related (GO id: 0006091), and photosynthesis and related processes (GO ids: 0015979; 0019684; 0022900; 0009767; 0055114). Similarly, hormone signal transduction of enriched KEGG pathway, correlated with the two-component- response-regulator-ORR9 like of GO enriched processes, response to abiotic (GO id: 0009628) temperature (GO id: 0009266) and cytokinin stimuli (GO id: 0009735).

In tuber as compared to shoot, plant hormone signal transduction of enriched KEGG pathway correlated with the serine/threonine protein kinases and receptor kinases of the GO enriched process i.e., response to biotic stimuli (GO ids: 0009607 and 0043207). The glucose-1-phosphate-adenylyl-transferase, glycogen-synthase, 1,4-α-glucan branching enzyme, glucan-water-dikinase-3, α-xylosidase, putative-granule-bound-starch-synthase, probable-α-glucosidase etc. of the GO enriched processes, i.e., cellular glucan- (GO id: 0006073; 0044042), xyloglucan- (GO id: 0010411) and cellular polysaccharide- (GO id: 0044264) metabolic processes and also cell wall organization/biogenesis (GO id: 0071554) matched well with starch, sucrose and galactose metabolism of KEGG pathway. Transporters like ABC transporter C of family member 3, 5 and 8; and ATP binding cassette transporter ABC2 of the GO enriched processes, i.e., response to biotic stimuli (GO id: 0009607; 0043207) matched with ABC transporter of enriched KEGG pathway. Similarly, mannitol dehydrogenases, peroxidases, strictosidine synthase like 5, leucoanthocyanidin dioxygenase like of the GO enriched process, i.e., response to biotic stimuli (GO id: 0009607; 0043207) correlated with enriched KEGG pathways like phenyl alanine metabolism, phenyl propanoid-, flavonoid- and indole alkaloid biosynthesis.

### PMN analysis

**Figure 3:**
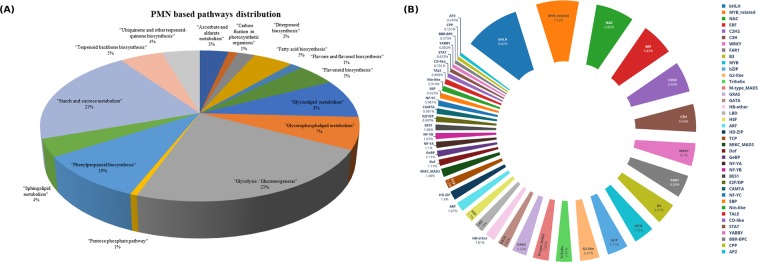
(**A**) Distribution of unigenes involved in different metabolite pathways based on PMN annotations. (**B**) Distribution of top 40 transcription factor families identified in *D. hatagirea*.

Unigenes of 15 metabolic pathways searched against Plant Metabolic Network (PMN) annotations revealed a total of 817 unigenes, wherein, glycolysis related genes (23%) followed by starch and sucrose metabolism (21%) and phenylpropanoid biosynthesis pathway (10%) were predominant (Fig. 3A; Fig. S9; Supplementary Table S7). The other important pathways included fatty acids, flavonoids and terpenoid backbone biosynthesis (5% each).

### Mining of transcription factors (TFs) for cold stress response

A total of 12,623 TFs belonging to 58 families were recorded. Among these, the bHLH family (known for its conserved but essential role in growth, development and jasmonate signaling) was predominant (9.87%). This was followed by MYB related TFs known for controlling cellular processes during stress response (7.62%) and NAC TFs (6.85%). Besides having regulatory roles in growth and development, the NAC TFs govern senescence and plant’s response to drought and chilling. TF families such as ERH (5.83%), C2H2 (5.46%), C3H, (4.96%), WRKY (4.1%), FAR1 (4.06%), B3 (3.87%) and MYB (3.72%) were the among the top 10 TF families (Fig. 3B; Supplementary Table S8).

### UHPLC-DAD confirmation of the presence of resveratrol and trans-stilbene in tubers

**Figure 4:**
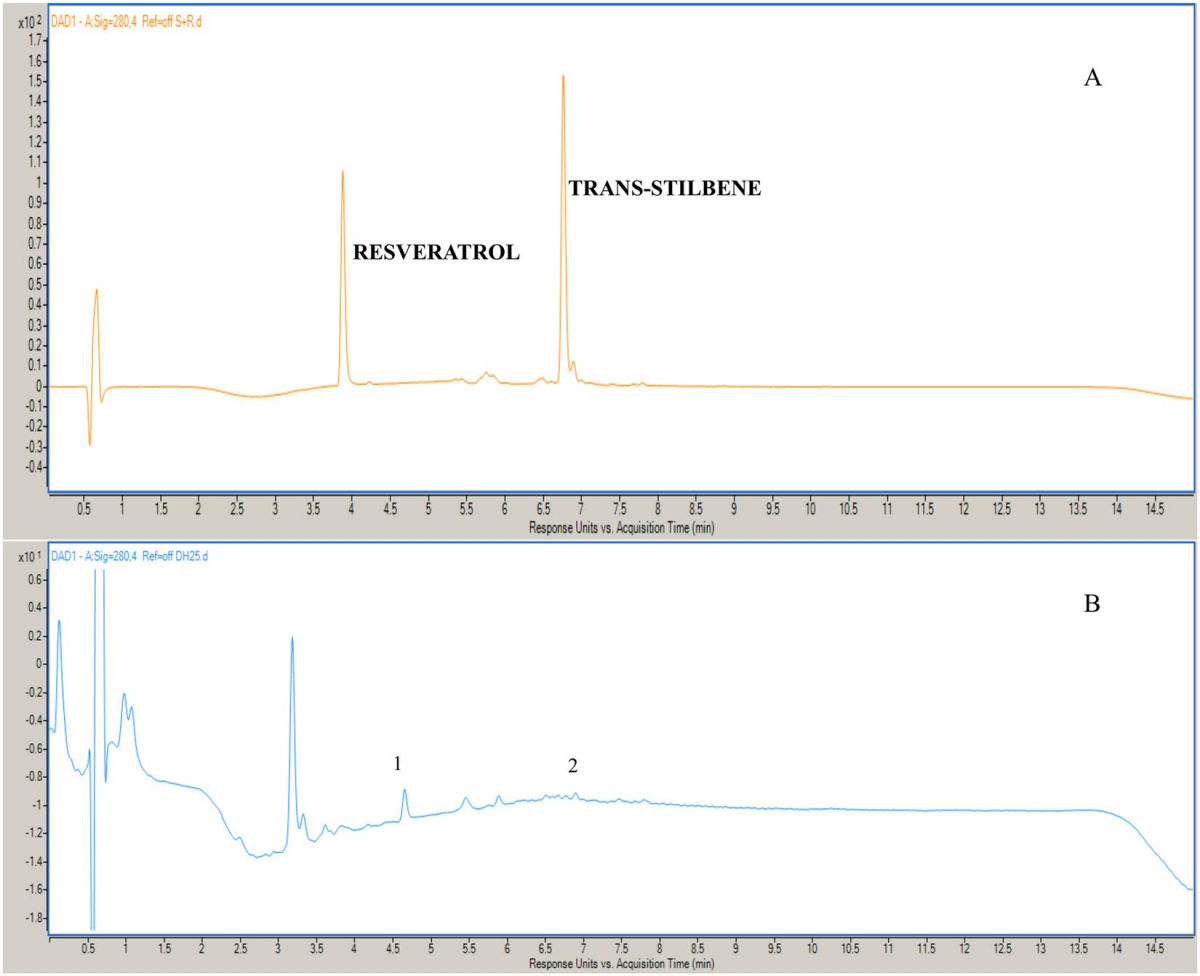
UPLC-MS chromatogram of (**A**) standards resveratrol and trans-stilbene (**B**) tuber sample of *D. hatagirea*, where 1 and 2 represent resveratrol and trans-stilbene.

UHPLC-DAD analysis of tuber tissue confirmed the presence of 3.21 µg/100 mg of fresh weight (f. wt.) of resveratrol and 2.49 µg/100 mg f. wt. trans-stilbene (Fig. 4).

### Temperature regulated phenological changes in *D. hatagirea*

**Figure 5:**
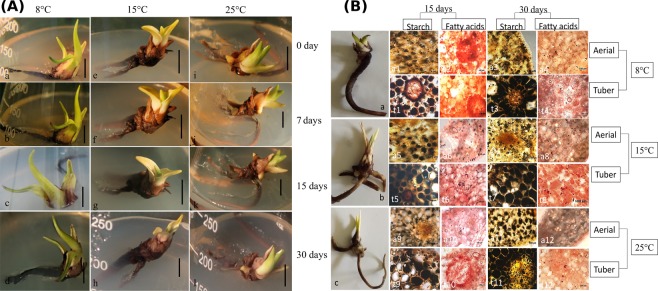
(**A**) Phenological response of *in vitro* plants of *D. hatagirea* to (a–d) 8 °C (e–h) 15 °C and (i–l) 25 °C after incubation for (a, e, i) 0 day (b, f, j) 7 days (c, g, k) 15 days and (d, h, l) 30 days. Scale bar = 1 cm. (**B**) Histochemical accumulation of starch and fats in (a1-a12) aerial and (t1-t12) below-ground parts of *in vitro* plants of *D. hatagirea* after 15 and 30 days at 8 °C (a1- a4; t1- t4), 15 °C (a5-a8; t5-t8) and 25 °C (a9-a12; t9-t12).

Differential phenotypic responses were recorded in clonal plants (developed through *in vitro* multiplication of protocorm-like-bodies) at 25, 15 and 8 °C. Distinct growth of both aerial and below ground parts was recorded at 25 °C (Fig. 5A). After 7 days at this temperature, there was new shoot bud initiation as well as tuber elongation. This growth progressed with time. After 7 days at 15 °C, there was only a slight increment in aerial growth but marked thickening of tuber. Tuber thickening and elongation of aerial parts progressed up to 15 days at this temperature. After 30 days, yellowing/senescence of aerial parts but remarkable thickening of tuber were recorded. At 8 °C also, yellowing of aerial parts but further growth of tuber were recorded after 7 days. Senescence of leaves initiated after 15 days but complete senescence occurred after 30 days. Tuber thickening ceased after 30 days at 8 °C.

### Temperature regulated histo-chemical changes in *in vitro* raised clonal plants of *D. hatagirea*

Differential accumulation of starch and fatty acids was recorded in tuber and aerial parts in response to temperature (Fig. 5B). Starch and fatty acids accumulation was invariably higher in the tuber as compared to leaves. Irrespective of tissues or temperature, accumulation of starch was highest after 30 days. When temperature was considered, starch and fatty acids accumulation was always higher in tubers at 8 °C followed by 15 and 25 °C. On the contrary, in aerial parts, starch accumulation was maximum at 25 °C followed by 15 and 8 °C.

### qPCR validation of DEGs obtained from RNA-seq data

**Figure 6:**
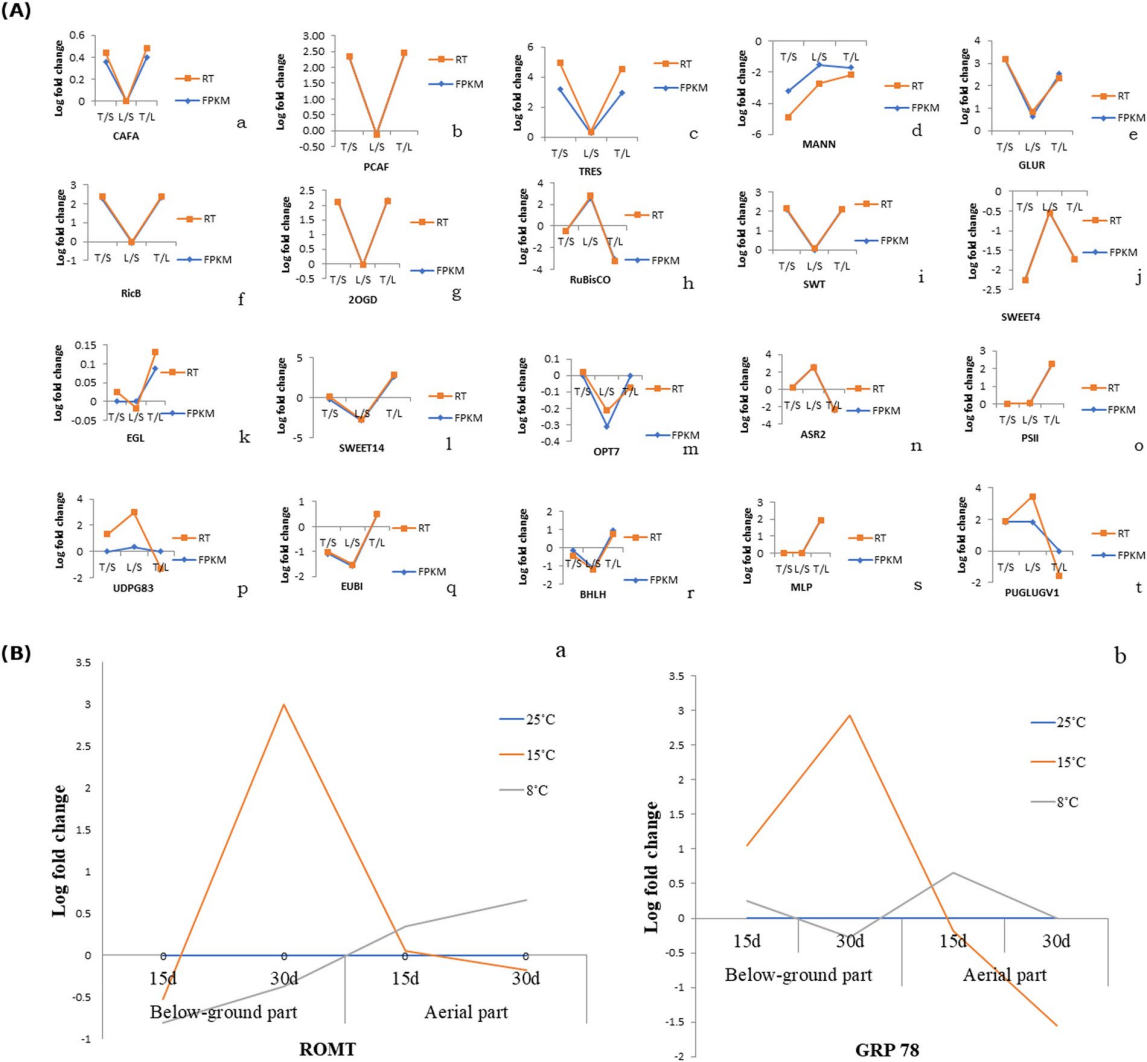
(**A**) qPCR showing differential expression of (a) caffeic-acid-3-O-methyltransferase-like (COMT), (b) putative-caffeoyl-CoA O-methyltransferase At1g67980 (CCOAMT), (c) trans-resveratrol-di-O-methyltransferase-like (ROMT), (d) mannan-endo-1,4-beta-mannosidase-6-like (MANN), (e) 78-kDa-glucose-regulated-protein-homolog (GRP78), (f) ricin-B-like-lectin R40C1 (RicB), (g) probable-2-oxoglutarate-dependent-dioxygenase AOP1 (2OGD), (h) LOW QUALITY PROTEIN: ribulose-bisphosphate-carboxylase-small-chain-clone-512-like (RuBisCO), (i) SWEET transporter 1 (SWT), (j) bidirectional-sugar-transporter-SWEET4-like (SWEET4), (k) EG45-like-domain-containing-protein-isoform-X2 (EGL), (l) bidirectional- sugar-transporter-SWEET14 (SWEET14), (m) oligopeptide-transporter-7-like (OPT7), (n) abscisic-stress-ripening-protein-2-like (ASR2), (o) photosystem II 22 kDa protein, chloroplastic (PSII); (p) UDP-glycosyltransferase-83A1-like, (q) E3 ubiquitin-protein-ligase-RHA2B-like (EUBI), (r) transcription-factor-bHLH111-isoform-X2 (BHLH), (s) MLP-like-protein-423 (MLP), (t) Putative-glucan-endo-1,3-beta-glucosidase-GVI (PUTGLUGV1) where T/S is tuber versus shoot, L/S is leaf versus shoot and T/L is tuber versus leaf. (**B**) qPCR expression of (a) trans-resveratrol-di-O-methyltransferase and (b) 78 kDa- glucose-regulated-protein-homolog gene in aerial and below-ground parts of *in vitro* plants of *D. hatagirea* after 15 and 30 days at 8, 15 and 25 °C (control).

From out of the top 50 DEGs obtained through RNA-seq of *D. hatagirea* plant parts, 20 were involved in photosynthesis, stress responses, sugar metabolism and secondary metabolite synthesis. qRT-PCR validation of these DEGs confirmed the reliability of the RNA-seq data because of their complete match with the expression patterns of 75% (15) of the selected genes. Statistically also, the correlation coefficient of RNA-seq data matched well with qPCR gene expression patterns (Fig. 6A). Interestingly, many of the validated genes are involved in phenylpropanoid pathway and also in resveratrol and stilbenes biosynthesis.

In qRT-PCR validation of ROMT and GRP78 genes, highest expression was recorded in below-ground parts of clonal plants treated for 30 days at 15 °C as compared to 25 °C (control). However, after 30 days, there was a marked down-regulation in gene expression in the aerial parts at this temperature. As compared to 15 °C, the expression of both ROMT and GRP78 genes were several folds lower in below ground parts at 8 °C, but several fold higher in the aerial parts, irrespective of days of treatment (Fig. 6B).

## Discussion

**Figure 7:**
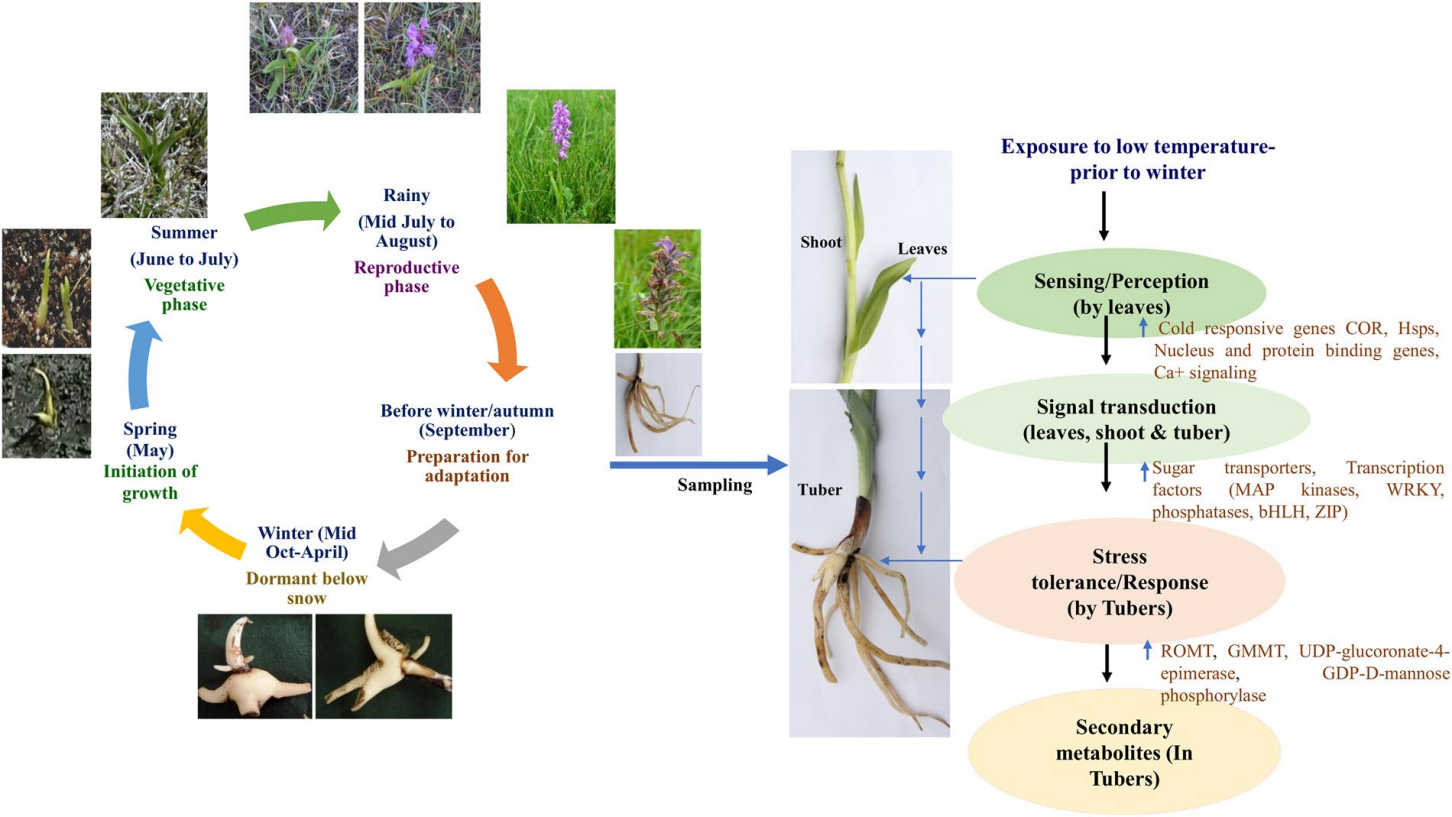
Graphical representation of phenological responses of *D. hatagirea* to seasons at alpine grasslands of Sach Pass in India and molecular basis of its advance preparation for winter.

**Figure 8:**
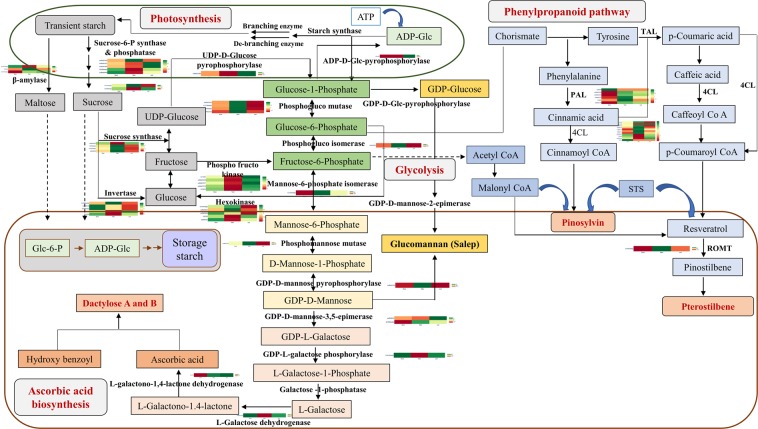
Probable mechanism of carbohydrate partitioning and secondary metabolite biosynthesis in *D. hatagirea* along with their differential gene expression in different plant parts further represented by heatmaps. Column 1, 2 and 3 represent shoot, tuber and leaf, respectively. Green and red color represent up-regulation and down-regulation, respectively. Detailed contigs list is given separately in supplementary Figure 10.

*D. hatagirea* is an endangered terrestrial orchid of alpine/subalpine Himalayas. Its tubers are reported to contain secondary metabolites like dactylorhin A, B, C, D, E; dactyloses A and B, millitarine and loroglossin^17,18^. While only dactylorhin B appears to account for its potent neuroprotective activity^19^, the various therapeutic properties of the plant have remained largely un-accounted for in traditional, Indian, Chinese and Tibetan systems of medicine. *De novo* transcriptome analysis was therefore, undertaken to gain insights into the secondary metabolite profile of *D. hatagirea*. A number of DEGs involved in the synthesis of various secondary metabolites were identified. Among these, glucomannan 4 phosphate mannosyl transferase (GMMT) and ascorbic acid biosynthetic enzymes such as GDP-D-mannose pyrophosphorylase were up-regulated in the tubers, thereby, accounting for a number of other therapeutic properties of the plant. Glucomannann, the major constituent of Salep in the tubers of this, and other medicinal orchids^3,10,20^ possess a range of medicinal properties^21^. Its biosynthesis is regulated by GMMT^22^. Ascorbic acid and p-hydroxy benzyl alcohol are the precursors of ‘dactylose A’ (1-deoxy-1-(4-hydroxyphenyl)-L-sorbose) and ‘B’ (1-deoxy-1-(4-hydroxyphenyl)-L-tagatose) biosynthesis via ascorbigen or (p-hydroxybenzyl)-3 keto hexulosonic acid lactone^23^. Additionally, the ROMT gene known for driving resveratrol biosynthesis^24^ was significantly up-regulated in the tubers; suggesting the presence of resveratrol and stilbenes in *D. hatagirea*. The compounds are reported to have anti-inflammatory, anticarcinogenic, cardioprotective, vasorelaxant, phytoestrogenic and neuroprotective properties. Stilbenes in particular, are valued for their efficacy against breast, lung, stomach, prostrate, pancreas, skin and colon cancers^25^. These properties are common to those reported for *D. hatagirea* in traditional system of medicine. Yet, the actual presence of resveratrol and trans-stilbene in *D. hatagirea* was unknown till date. Our transcriptome analysis and PMN revealed the synthesis of (i) malonyl CoA through glycolysis, (ii) *p*-coumaric acid via shikimic acid pathway and (iii) cinnamyl CoA via phenylpropanoid pathway in *D. hatagirea*. These pathways contribute towards the direct biosynthesis of stilbenes^24^. The caffeic and coumaric acids, and caffyeol CoA from the shikimate pathway also contribute towards the synthesis of resveratrol and consequently, stilbenes (Fig. 7 and S10; Supplementary Table S9). Moreover, the DEGs of GDP-D-mannose biosynthesis from glucose-6-phosphate were recorded in tuber versus shoot. Since glucose-6-phosphate is the common precursor for both glucomannan and glucosides biosynthesis, it was hypothesized that once GDP-D-mannose is biosynthesized, *D. hatagirea* has the option of synthesizing a host of other metabolites for resilience against stress. Synthesis of glucomannans for the fortification of perennating tubers against cold stress in one route; and biosynthesis of dactylorhins and dactyloses via ascorbic acid, the key intermediate for strengthening of antioxidant capacity in the second route are good examples (Fig. 8). Furthermore, integration of GO enriched processes and KEGG pathways revealed the enrichment of (i) strictosidine synthase responsible for the synthesis of indole alkaloids; (ii) zeaxanthin-dioxygenase and prolylycopene isomerase responsible for the synthesis of carotenoids and (iii) 2-carboxy-1,4-naphthoquinone phytyltransferase responsible for phylloquinone (vitamin K) biosynthesis. Indole alkaloids are valued for their anti-hypertensive, anti-depressent and anti-cancer properties^26,27^, whereas, carotenoids are popular for various health benefitting effects^28,29^. The above metabolites account for the various therapeutic properties of *D. hatagirea* that had remained unaccounted for till date.

Freezing stress is the all pervading factor governing all life forms in alpine habitats. Herbs inhabiting such regions are generally opportunistic and prefer to avoid, rather than tolerate the freezing air temperatures of winter. *D. hatagirea*, the alpine Himalayan herb of Sach Pass in India was also found to avoid freezing stress. This was evident from *in vitro* studies, where both aerial and below-ground parts of *D. hatagirea* grew actively at 25 °C (representing the average day temperature during summer at its habitat niche). However, yellowing of aerial parts with biased growth of tuber was recorded at 15 and 8 °C, representing the average day temperatures during the beginning and end of September, respectively (Fig. 5A). Opportunistic plants maximize their photosynthetic efficiency during summer for rapid formation of new structures because much before winter, organs of perennation have to be ready to receive photosynthates from aerial parts^30^. As air temperatures decline, the plants of such habitats translocate all their photosynthates to below-ground parts for their growth, energy, conversion into storage forms, secondary metabolite synthesis and stress tolerance^31^. Thereafter, the plants begin to shed their costly and difficult to maintain aerial parts. The enrichment of *in situ* leaf β-galactosidase in GO processes and KEGG pathway coupled with senescent leaves, maximally thick tubers having high starch accumulation and highest qRT PCR expression of ROMT and GRP78 genes (Fig. 6B) confirmed cold avoidance in *D. hatagirea* growing *in vitro* at 15 °C. β-galactosidase activity is at peak during terminal phase of leaf senescence^32^.

Much before the onset of winter, the leaves followed by aerial parts of cold avoiding plants sense the signals of declining air temperatures and then transduce them to different parts^33^. The plants respond to these signals by beginning an advance participation in freezing tolerance mechanism for survival during winter^33^. Highest number of cold responsive DEGs were therefore, up-regulated in leaves as compared to tubers of *D. hatagirea* plants collected during September. The month signifies the end of favourable season for aerial growth of herbs inhabiting Sach Pass, and also a period when the quantity of light available for photosynthesis reduces significantly before the onset of freezing winter. Hence, 53 and 20 DEGs of various signaling pathways were up-regulated in leaves as compared to tuber and shoot, respectively. There was also a predominance of cold stress responsive DEGs in all plant parts of *D. hatagirea* in GO function categories; with 60 DEGs of cold stress perception and response in shoot versus leaf; 50 in shoot versus tuber; and 79 in tuber versus leaf. DEGs related to response to environmental and hormonal stimuli, photosynthesis and related processes, cell wall biogenesis/organization, glucan metabolism and transport were significantly enriched in GO enrichment and KEGG pathway (Supplementary table S6). For example, the photosynthesis antenna protein, LHCII was enriched in the leaves. The protein is known to maintain the redox balance between PSI and PSII when light available for photosynthesis in nature reduces suddenly^34^. Additionally, calcium signaling and ion binding proteins, MAP kinases, phospholipases and phosphatase families, receptor kinases, hormone-signaling and histidine-containing-phosphotransfer-proteins and transcription factors such as WRKY, bHLH and bZIP were differentially expressed in leaves as compared to shoot and tuber (Supplementary Tables S8 and S10). After perception and signal-transduction of cold/freezing stress; sequential phosphorylation and activation of proteins involved in calcium-ion-binding, membrane-ion-channels and two-component-response-regulators help a plant to complete its stress tolerance response^35–37^. The MAP kinases in particular, act downstream of sensor/receptor cascade to facilitate post-translational phosphorylation during cellular responses to freezing stress^38–40^, while also imparting abiotic stress tolerance^41^. Water-stress-responsive DEGs including ABA-inducible-protein-PHV-1 like and protein-proton-gradient-regulation were up-regulated in leaves as compared to tuber and shoot, probably to combat drought stress. Freezing being associated with drought stress cause progressive desiccation of aerial parts^42^.

With decline in air temperatures at the end of favourable season, cold avoiding plants first translocate their aerial metabolites into below-ground tubers and then prepare to shed all aerial parts^31^. The entire process requires active break down of photosynthates (starch) into sucrose and other sugars like maltose, triose-phosphates and finally, glucose in the ‘source or the leaves’ for translocation out of leaves to the ‘sink or below ground parts’^30^. Hence, there was up-regulation of mainly, isoamylase, β-amylase, triose-phosphate isomerase, β-galactosidase, transporters and hexo-, phospho-, phosphofructo- glycerate- kinases in leaves as compared to shoots of *D. hatagirea*. Phosphofructokinase partitions sucrose/starch in leaves for regulation of fructose-biphosphate levels^43^, whereas, hexose phosphates formed after starch degradation are transported out of chloroplast, and finally out of leaves^44,45^. Thus, DEGs of sucrose synthase, sucrose phosphate synthase, bidirectional-sugar-transporter-sweet-14 and 1a-like, the sugar-transporter- ERD6-like and their isoforms were up-regulated in the leaves of *D. hatagirea*. The uniporter SWEETs are sugar transport proteins that help in translocating sugars down a concentration gradient^46^.

Shoots are the major translocators of photosynthates, nutrients and water to different sink tissues. They also participate in signal transduction, photosynthesis, defence and structural support. Thus, quite a high number of DEGs (1239) were up-regulated in shoot versus leaf including those of cold response, calcium signaling and ion binding, photosynthesis, starch and sucrose biosynthesis, glycolysis, sucrose catabolism, ABA and water deficit; and also lipids, phenylpropanoid and flavonoid biosynthetic pathways.

All throughout the freezing winter, the perennating tubers of alpine herbs lie buried under the snow cover because the temperatures are comparatively more favorable there^26^. Much before winter, the tubers become ready to import sugars from the sieve tubes and companion cells of stem phloem and start serving as final sinks. Thus, DEG of glycerate kinase was up-regulated in tuber versus leaf. Glycerate kinase regulates the flow of carbon towards sucrose synthesis^47^. A portion of the imported sugars is utilized for fortification and maintenance of the tubers under snow cover. A good part of it is also diverted towards secondary metabolites synthesis^48^. A major part is also maintained as such for mobilization and support of new growth during following spring^19^. In this regard, DEGs of β-glucosidase, trehalose phosphatase, GMMT, glucuronate 4 epimerase; and those involved in signal-transduction, phytohormone activation, metabolite-synthesis, cell-wall-remodeling, lignification, carbohydrate synthesis and stress tolerance were up-regulated and enriched in *D. hatagirea* tuber as compared to shoot. Water dikinases regulate the growth of storage roots and also facilitate β-amylase mediated conversion of starch into maltose and other transient forms during early stages of cold shock^49^. Thus, a glucan water dikinase (GWD3) was enriched. Moreover, DEGs of glycerolipids, glycerophospholipids, sphingolipids, β-glucosidases, glucose-1-phosphate-adenyl-transferase (AGP2), glycogen synthase, trehalose-6-phosphatase (T6P) and the heat shock protein, GRP78 were up-regulated and/or enriched in tubers. T6P governs plant growth and development based on the sucrose status of a tissue at a given time^50^, whereas, trehalose obtained after de-phosphorylation of T6P stabilizes cell membranes during freezing stress. AGP2 participates in starch and sucrose metabolism in response to environmental stress^51^, whereas, glycogen synthase regulates the type and level of carbohydrate signals^52^.

All the above reveal an advance preparedness of *D. hatagirea* in September for survival during freezing winter.

## Conclusion

The study reports the transcriptome of *D. hatagirea* for the first time. The tissue specific differential expression of unigenes in the plant collected during September indicates its participation in the freezing tolerance mechanism much before the onset of winter. Based on these findings, probable biosynthetic routes for the synthesis of important secondary metabolites and a hypothetical mechanism on how *D. hatagirea* responds and prepares for oncoming freezing stress through carbohydrate metabolism and diversion of photosynthates towards secondary metabolite synthesis are proposed. Confirmed presence of appreciable amounts of resveratrol and trans-stilbene, two high value secondary metabolites and specific indication of several others in the tuber of *D. hatagirea* are also being reported for the first time. The study paves the way for genetic manipulation of important secondary metabolites. It also confirms the temperature dependent opportunistic growth behavior of this cold avoiding terrestrial alpine orchid. The environmental cues (temperature and light) identified through *in vitro* studies and GO enrichment data provide useful leads for modulating the identified metabolites in the plant. The study also contributes towards sustainable utilization and conservation of this high value endangered medicinal orchid.

## Materials and Methods

### Documentation of seasonal and vegetation characteristics at natural habitat of *D. hatagirea* for plant sample collection

The different seasons prevailing at the region below Sach Pass, Chamba, Himachal Pradesh, India, the average day temperatures prevailing at the region during these seasons and the growth behaviour of the herbs inhabiting the region were documented on the basis of available reports, and also interactions with researchers who visited the place for surveys and plant collection. Based on this information, *D. hatagirea* plants growing below Sach Pass were collected during September, the month signifying the period before the onset of freezing winter. The plants thus, collected served as the source material for the present study.

### RNA library preparation

The iRIS method of Ghawana *et al*.^53^ was used to extract total RNA from leaf, shoot and tuber of the plant collected from region below Sach Pass. RNA samples from each plant part were taken in triplicates. The quality and quantity of the total RNA were determined using Nanodrop 1000 spectrophotometer (Thermo Fisher Scientific, USA) and integrity by the Bioanalyzer RNA nanochip (Agilent 2100 technologies, USA).

### Transcriptome sequencing

High quality RNA (5.0 µg) from each plant sample was isolated in triplicates and TruSeq RNA sample Prep Kit v2 (Illumina Incorporation, USA) was used for RNA library preparation. The libraries were quantified using Qubit^TM^ RNA/Qubit dsDNA BR assay kits in a Qubit 2.0 Fluorometer (Life technologies, USA). The insert size of the library was verified using a bioanalyzer DNA 1000 chip (Agilent 2100 technologies, USA). The prepared library (10 pM) was loaded onto the flow cell using TruSeq PE Cluster Kit v5 on cluster station (Illumina Incorporation, USA), and the clonally amplified clusters analyzed using a Genome Analyzer IIx (Illumina, USA) for paired-end (PE) (2 × 72) sequencing. The raw sequencing data were then transformed into single end (SE) 72 bp reads by the GERALD base-calling (a CASAVA package of Illumina) and the resulting sequence reads were stored in FASTQ format. The quality of the sequence reads obtained was checked using Fast QC and further processed to remove adapter sequences, low quality reads and reads of very short length by initial filtering. The initial filtering was done using the in-house developed tool filteR^13^. Vertical quality filtering was performed to ensure position based consistency of quality score in read files, whereas, horizontal filtering was used to confirm the quality of each read and trim adapters. For quality filtering, phred QV30 cut-off was used for 70% bases of a read. Reads that failed to fall under cutoff category were discarded. Remaining high quality reads were used for *de novo* assembly.

### *De novo* sequence assembly

*De-novo* assembly of the transcriptomes was performed using SOAPdenovo-Trans-127mer with default parameters, which uses de brujin graph algorithm. The quality of the transcripts was ensured by trimming the sequence reads and removing the sequencing adapters. As the read length was 72 bases, varying lengths of 21 to 69 kmer and average insert size of 200 bp of paired end reads were used during assembly. The same information was used for scaffolding. Out of the varying kmer length based assembly, a final assembly was selected on the basis of different statistical parameters like N50, average contig length etc. The final assembly was further improved using different clustering based approach. TGICL-CAP3 was used with terminal length of 40 bp and 90% identity for terminal joining. Resultant merged contigs and singleton contigs were used as inputs for CD-HIT clustering. To reduce the redundancy between the same type of contigs, CD-HIT-EST was used with 95% similarity cut-off. For further improvement, a well established approach of dissimilar clustering (DS clustering) was implemented^13^. A set of in-house developed python scripts was used to DS cluster contigs that did not have any sequence similarity but belonged to different regions of the same gene. Those contigs which did not have any significant hit were further converted into six frame ORFs and searched against the conserved domain database (CDD) using rps blast.

### Functional annotation of the assembled transcriptome and classification

In order to identify and assign biological functions to each gene, the transcriptomes of leaf, shoot and tuber obtained after filtration were annotated using BLASTx similarity search against NCBI-nr database with an E-value cut-off 10^−05^, as practiced in our earlier report^13^. Databases like Uniprot, GO, KEGG and NCBI-nr were used for homology searches. Multiple hits were reported by Annot8r for maximum unigenes. The best hits were selected for each unigene on the basis of highest bit score and E-value. Most of the GO, KEGG and EC based annotations were done using Annot8r.

### Differential gene expression analysis in plant parts of *D. hatagirea*

DEGs of leaf, shoot and tuber of *D. hatagirea* were analyzed. To quantify the expression of unigenes, reads for each plant part (condition) were separately mapped onto the unigenes using Bowtie with default parameter of 2 mismatches and insert size of 200 bp. Counts of unique read mapping were calculated using in-house developed perl script. The count files were applied to edegR as matrix of mapped reads count for each condition. One to one analysis was performed between three different plant parts. To select differentially expressed genes, p-value < 0.05 cutoff was used. Contigs having Log FC >2 along with p-value <  = 0.05 were considered as significant differentially expressed contigs. Count files were converted into FPKM using in-house scripts in order to calculate the expression of each replicate. Associated GO, KEGG and EC annotations along with expression value in terms of FPKM were parsed using in-house developed shell scripts. Three different comparisons between three different conditions were generated using ggplots, Biobase and cluster package in R for all the differentially expressed genes. Tissue specific differentially expressed genes are represented as Venn diagrams. Heatmaps of different genes involved in cold stress related processes leading to the synthesis and accumulation of secondary metabolites were generated.

DEG’s of different tissues were also subjected to singular enrichment analysis using AgriGO (http://systemsbiology.cau.edu.cn/agriGOv2/c_SEA.php). GO enrichment analysis was performed using Hygrogeometric test with Bonoferroni analysis method and p-value ≤ 0.05. Finally, the data obtained from enrichment of GO annotations and KEGG pathways were correlated and integrated for identification of discrepancies and similarities in the two analyses. Furthermore, unigenes were categorized on the basis of their functional roles in metabolic networks. Enzyme classification of these was searched in Plant Metabolic Network (PMN).

These unigenes were also blasted against Plant TFDB for mining the transcription factors and the top hits were applied.

### Validation of DEGs using quantitative PCR

Quantitative real time PCR (qPCR) was performed as per StepOne Software v2.3 (Applied Biosystems, Thermofisher Scientific, USA) for validation of genes identified through RNA-seq. Total RNA was isolated from each plant parts in triplicates. These were used for the synthesis of cDNA by the Verso cDNA synthesis kit. Based on fold change and biological functions, top genes were selected. DEG specific primers were designed using the Primer Express 3.0 software (Supplementary Table S11). The power SYBR Green PCR Master Mix (Applied Biosystems, USA) was used. All reactions were performed in triplicates using Applied Biosystems StepOne real time PCR (Thermofisher Scientific, USA). The qPCR program comprised of a pre-incubation step of 10 min at 95 °C, 40 cycles of DNA amplification for 15 seconds at 95 °C and 1 min at 60 °C. This was followed by a melting-curve program comprising of 95 °C for 15 seconds, 60 °C for 1 min and 95 °C for 15 seconds. Gamma tubulin complex component 4 served as control. The data obtained through qPCR were analysed by comparative Ct method for comparison of DEGs between plant parts.

### Confirmation of the presence of resveratrol and trans-stilbene in tubers through UHPLC-DAD analysis

Fresh samples of tuber tissue (100 mg) were homogenized in liquid nitrogen using a pestle and mortar. The homogenate was first extracted with 2.0 ml, and then twice with 1.5 ml of 70% methanol. The extract was centrifuged at 8000 rpm for 10 min at room temperature. The supernatants were pooled and final volume was made up to 5.0 ml with 70% methanol to serve as sample. The standards and samples were subjected to UHPLC-DAD analysis.

### UHPLC-DAD analysis

The LC separation was performed using hydrophilic interaction chromatography with an Acquity UPLC BEH C18 column (2.1 × 50 mm, 1.7 µm, Agilent Technologies) operated by Agilent 1290 Infinity II UHPLC system (Agilent, Santa Clara, USA). The mobile phase consisted of A (water containing 0.1% formic acid) and B (acetonitrile containing 0.1% formic acid) and was run in gradient elution i.e., 0–4 min, 90% B; 4–11 min, 90% B and 9–15 min, 10% B. The flow rate and injection volumes were 0.25 ml/min and 2 µl, respectively. Elution was monitored at 280 nm by using a PDA detector and column temperature was maintained at 30 ®C. Detection of resveratrol and trans-stilbene was performed by spectral matches with standards of resveratrol and trans-stilbene (Sigma-Aldrich) prepared in methanol (1.0 mg/ ml; w/v).

### Effect of temperature on the phenology and growth behavior of *in vitro* raised clonal plants

Clonal plants of *D. hatagirea* were raised *in vitro* through multiplication of protocorm-like-bodies on MS^54^ medium supplemented with 2.5 mg/l meta-topolin, 3.0% sucrose (w/v) and 0.76% agar (w/v) at pH 5.8. After two sub cultures of 60 days each, 2.0 cm long plants were incubated for 30 days at 25, 15 and 8 °C under 16 h light (25 µmol m^−2^ s^−1^ from Philips white fluorescent tubes) and 8 h dark. While 25 °C served as control, representing average day temperature during summer; 15 and 8 °C represented average day temperatures during early and late September at Sach Pass. Visual observations on the phenology of *in vitro* plants incubated at 8, 15 and 25 °C were recorded at 7 days interval. Histochemical analyses of starch and fats was also carried out after 15 and 30 days. For this, hand sections of leaf and tuber were cut and stained using iodine solution (0.3% iodine and 1.5% potassium iodide in water) for starch and 0.5% Sudan III in 70% ethanol for fats. After incubation for 20 min, the sections were washed using 50% alcohol and observed under a fluorescent microscope (Axio Imager, M1, Carl Zeiss, Gmbh, Germany).

### Temperature regulated expression of GRP78 homolog and ROMT in *in vitro* raised clonal plants

Total RNA was isolated in triplicates from aerial (leaves and shoot) and below-ground (tuber and roots) parts of *in vitro* raised clonal plants growing at 8, 15 and 25 °C for 15 and 30 days. cDNA was synthesised from these samples by the Verso cDNA synthesis kit. These were subjected to qPCR using primers specific to (i) the heat shock protein 78 kDa glucose regulated protein homolog (GRP78), and (ii) resveratrol O methyl transferase (ROMT) responsible for the synthesis of stilbenes from resveratrol. The qPCR cycles optimized for top 15 genes were also used for GRP78 and ROMT. The gamma tubulin complex component 4 served as internal control. The results of the qPCR data were analysed by comparative Ct method.

## Supplementary information

Supplementary Information

Supplementary Table S4

Supplementary Table S5

Supplementary Table S6

Supplementary Table S7 to S11
