# Supplementary material for: De novo transcriptome provides insights into the growth behaviour and resveratrol and trans-stilbenes biosynthesis in Dactylorhiza hatagirea - An endangered alpine terrestrial orchid of western Himalaya
Source: Sci Rep. 2019 Sep 11;9:13133. doi: 10.1038/s41598-019-49446-w (PMC6739469; doi:10.1038/s41598-019-49446-w)
Supplement: Supplementary file 1 — Supplementary Information [file 41598_2019_49446_MOESM1_ESM.pdf]

Supplementary Information:

Original Research Article:

De novo transcriptome provides insights into its growth behaviour and resveratrol and trans-stilbenes biosynthesis in *Dactylorhiza hatagirea* - An endangered alpine terrestrial orchid of western Himalaya

Nisha Dhiman[1,2#], Nitesh Kumar Sharma[1,2#], Pooja Thapa[1#], Isha Sharma[1], Mohit Kumar Swarnkar[1], Amit Chawla[1,2], Ravi Shankar[1,2*] and Amita Bhattacharya[1,2*]

[1]Division of Biotechnology, CSIR-Institute of Himalayan Bioresource Technology, Palampur-176061, H.P., India

[2]Academy of Scientific and Innovative Research, Human Resource Development Centre, Ghaziabad-201 002, Uttar Pradesh, INDIA

For correspondence: amitabhatta@ihbt.res.in; amitabhatta@yahoo.co.uk; ravish@ihbt.res.in

#Equal authorship

*Corresponding author

Amita Bhattacharya, Ph.D.
Division of Biotechnology,
CSIR-Institute of Himalayan Bio-Resource Technology,
Palampur, India.

E-mail address: amitabhatta@yahoo.uk.co.in and ORCID ID: orcid.org/0000-0003-1936-2995

Supplementary figures:

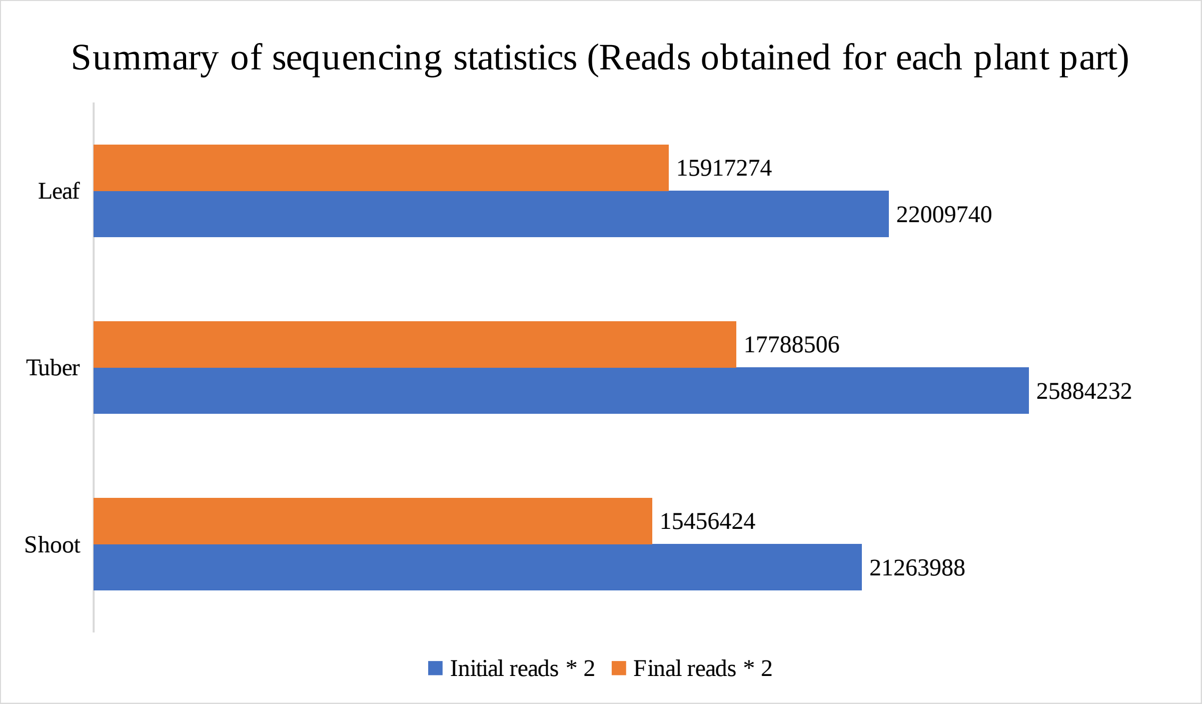

Supplementary Figure S1: Summary of sequencing statistics showing reads obtained for each plant part

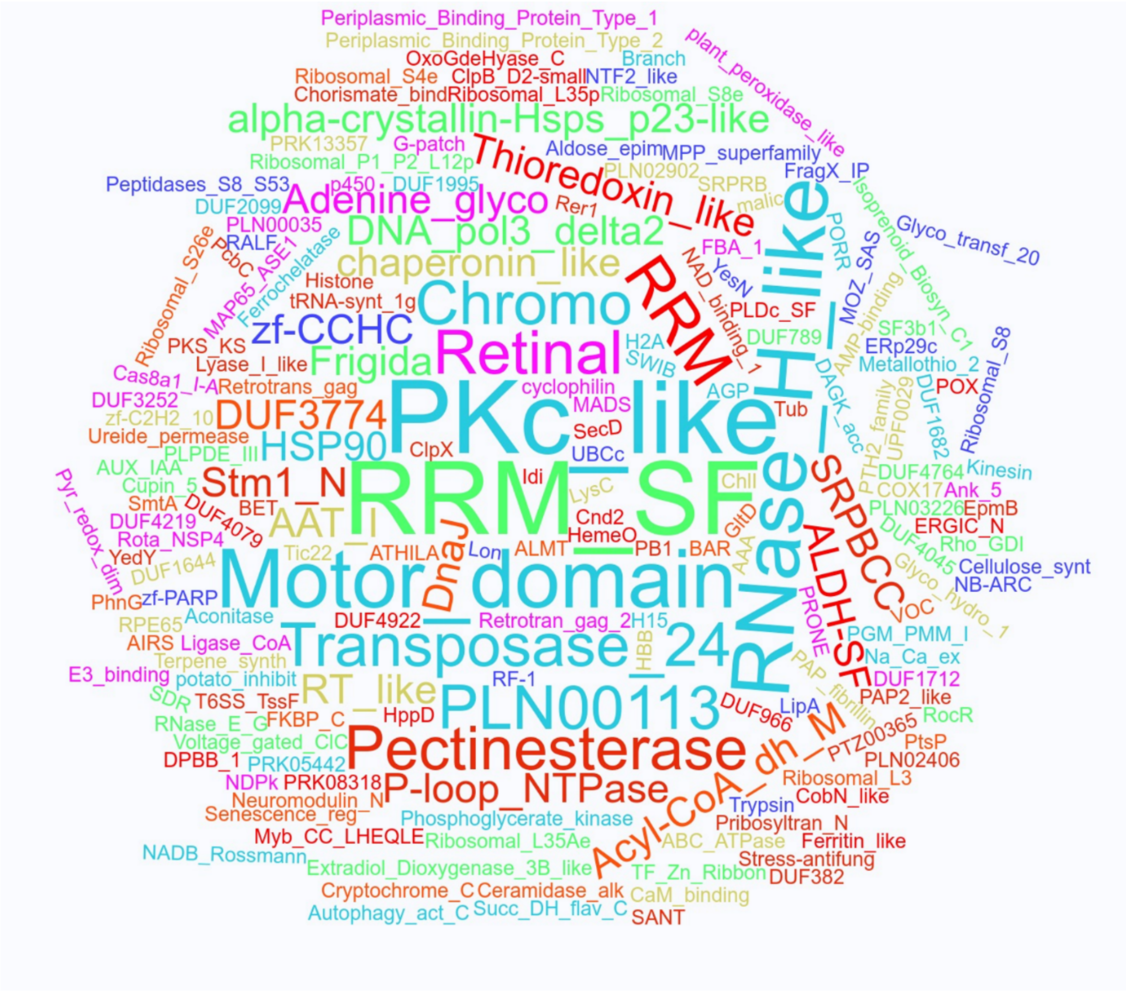

Supplementary Figure S2: RPS blast against CDD database showing RRM_SF and Pkc_like maximally enriched term

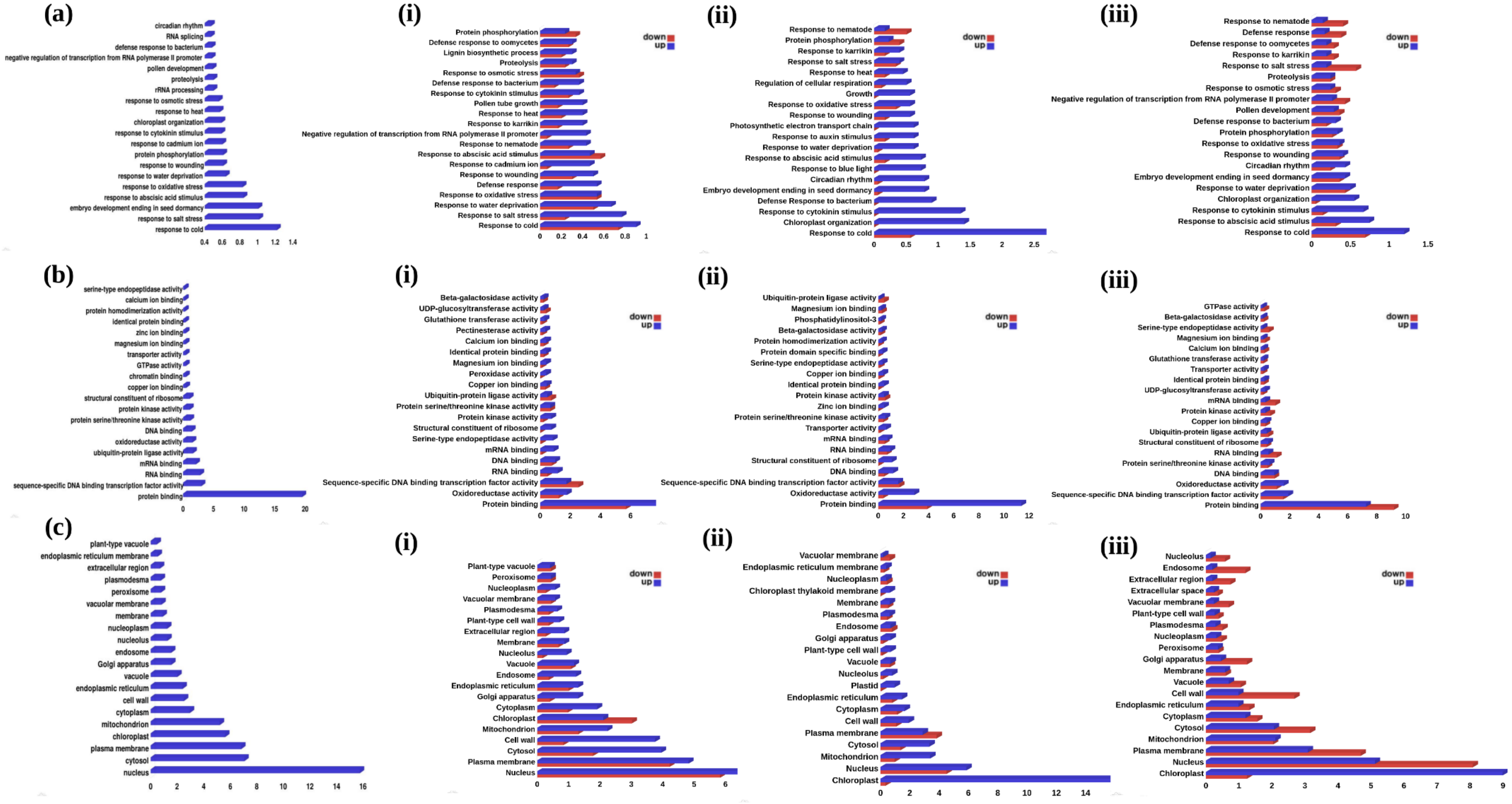

Supplementary Figure S3: Pooled datasets showing distribution of unigenes in *D. hatagirea* across GO classification (a) biological, (b) molecular and (c) cellular process categories; where (i-iii) represent differential expression in (i) shoot-versus-tuber, (ii) shoot-versus-leaf (iii) tuber- versus-leaf

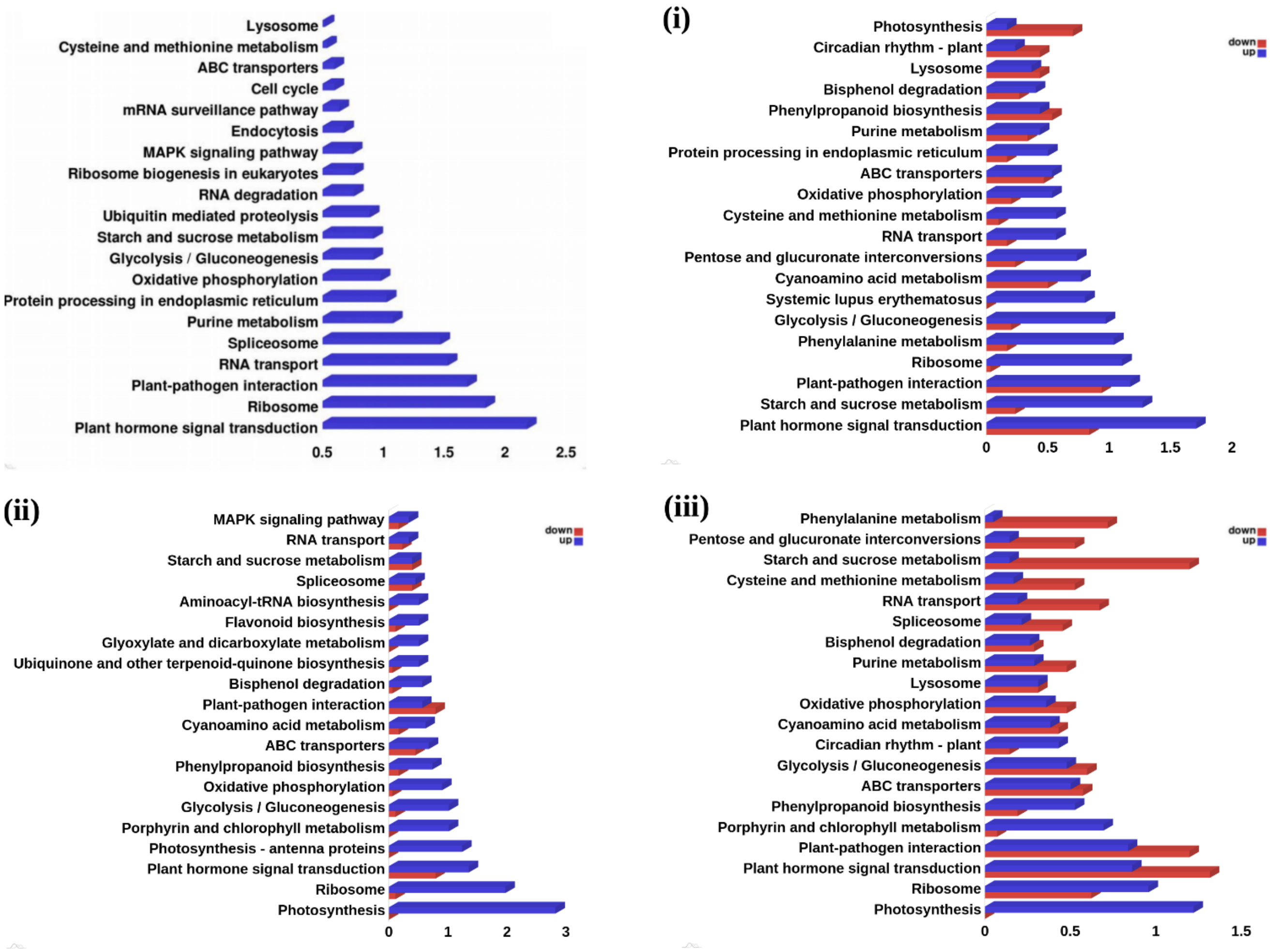

Supplementary Figure S4: Pooled datasets showing distribution of unigenes in *D. hatagirea* across KEGG pathways where (i-iii) represent differential expression in (i) shoot-versus-tuber, (ii) shoot-versus-leaf (iii) tuber- versus-leaf

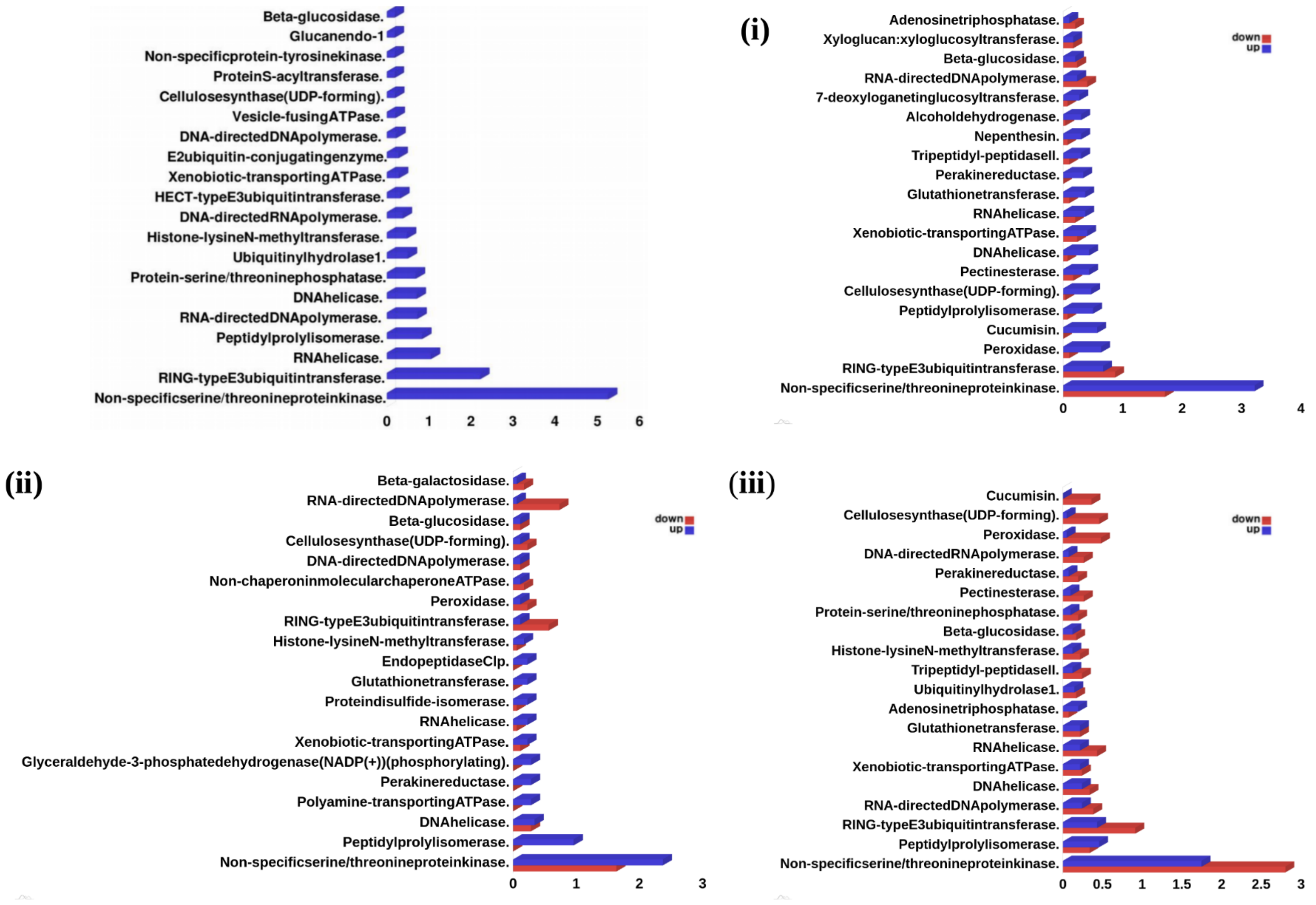

Supplementary Figure S5: Pooled dataset showing the distribution of unigenes in *D. hatagirea* across various categories of enzyme classification pathways where (i-iii) differential expression in (i) shoot-versus-tuber, (ii) shoot-versus-leaf (iii) tuber- versus-leaf

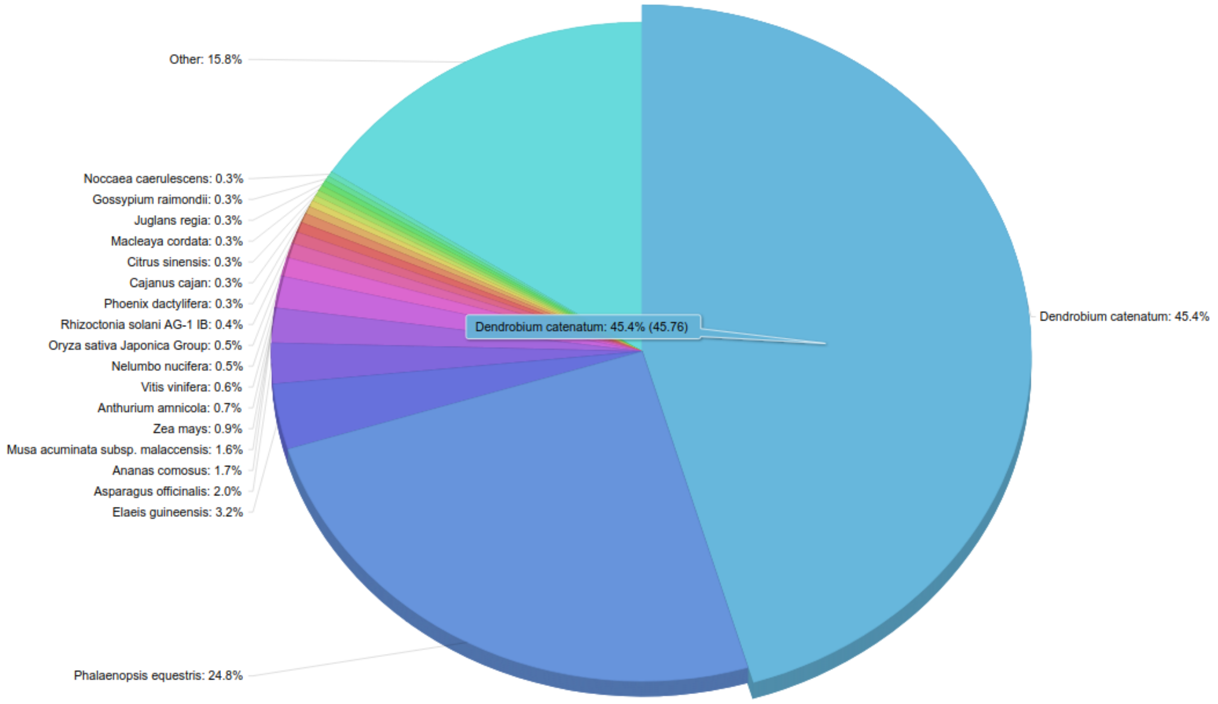

Supplementary Figure S6: Distribution of unigenes in *D. hatagirea* across various species on the basis of blast similarity search

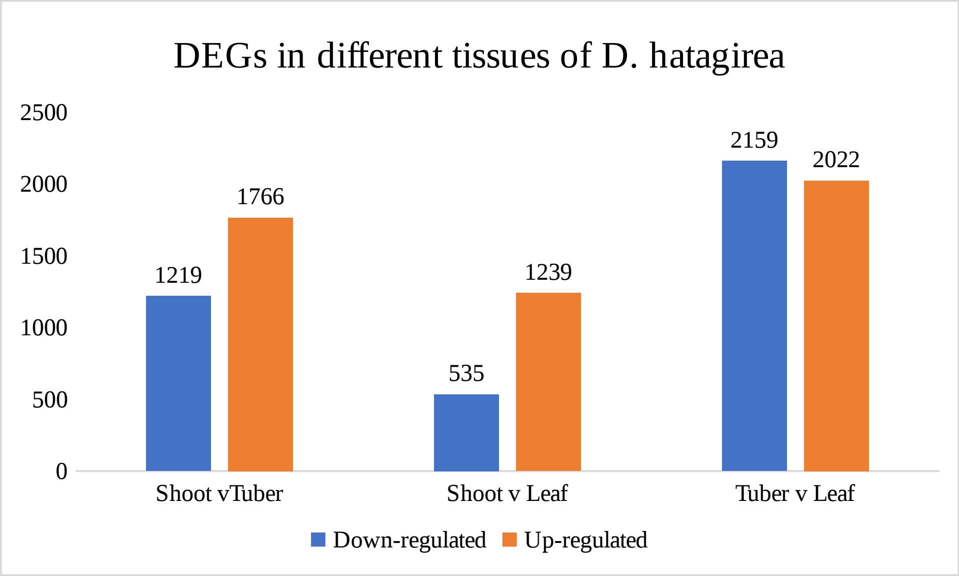

Supplementary Figure S7: Bar graph showing total number of DEG's (log fold change ≥ 2) in different plant parts (a) down-regulated (b) up-regulated

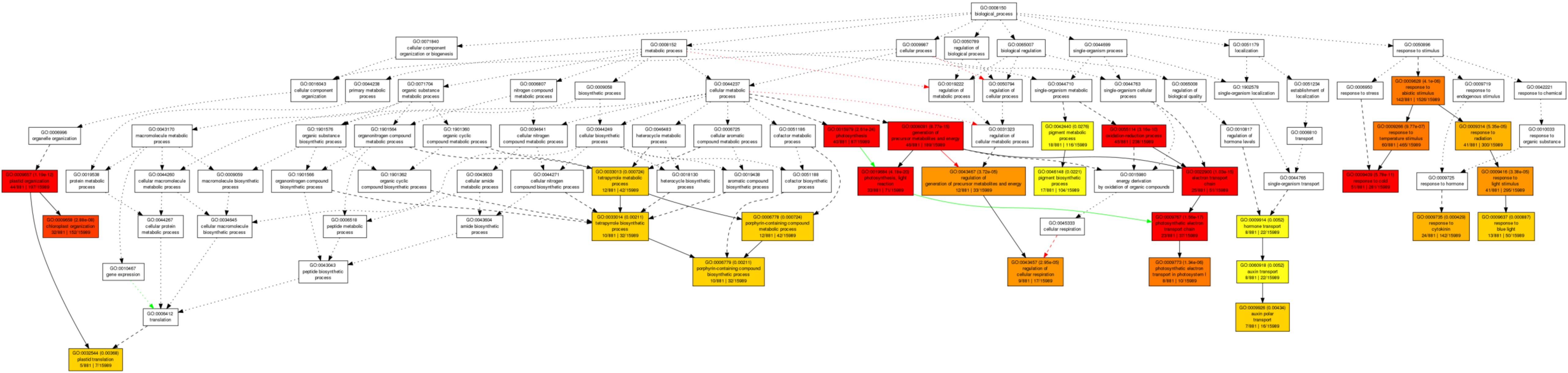

Supplementary Figure 8(A)

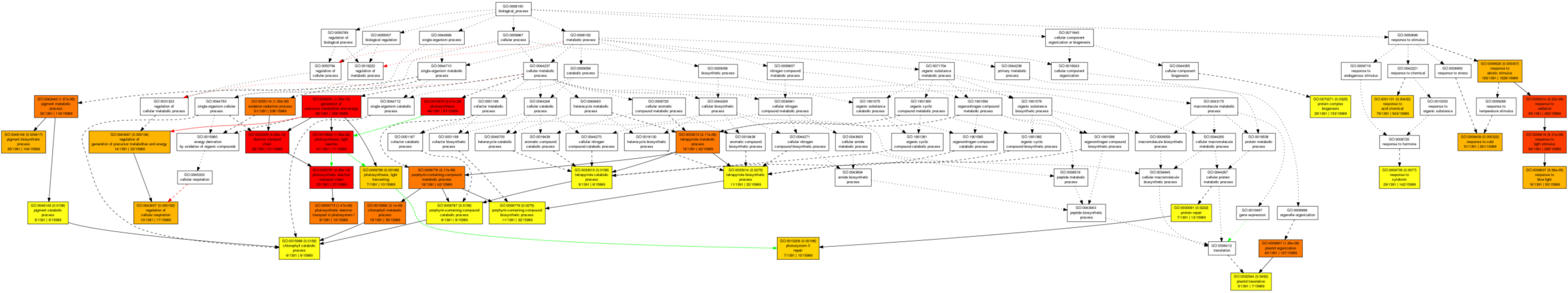

Supplementary Figure 8(B)

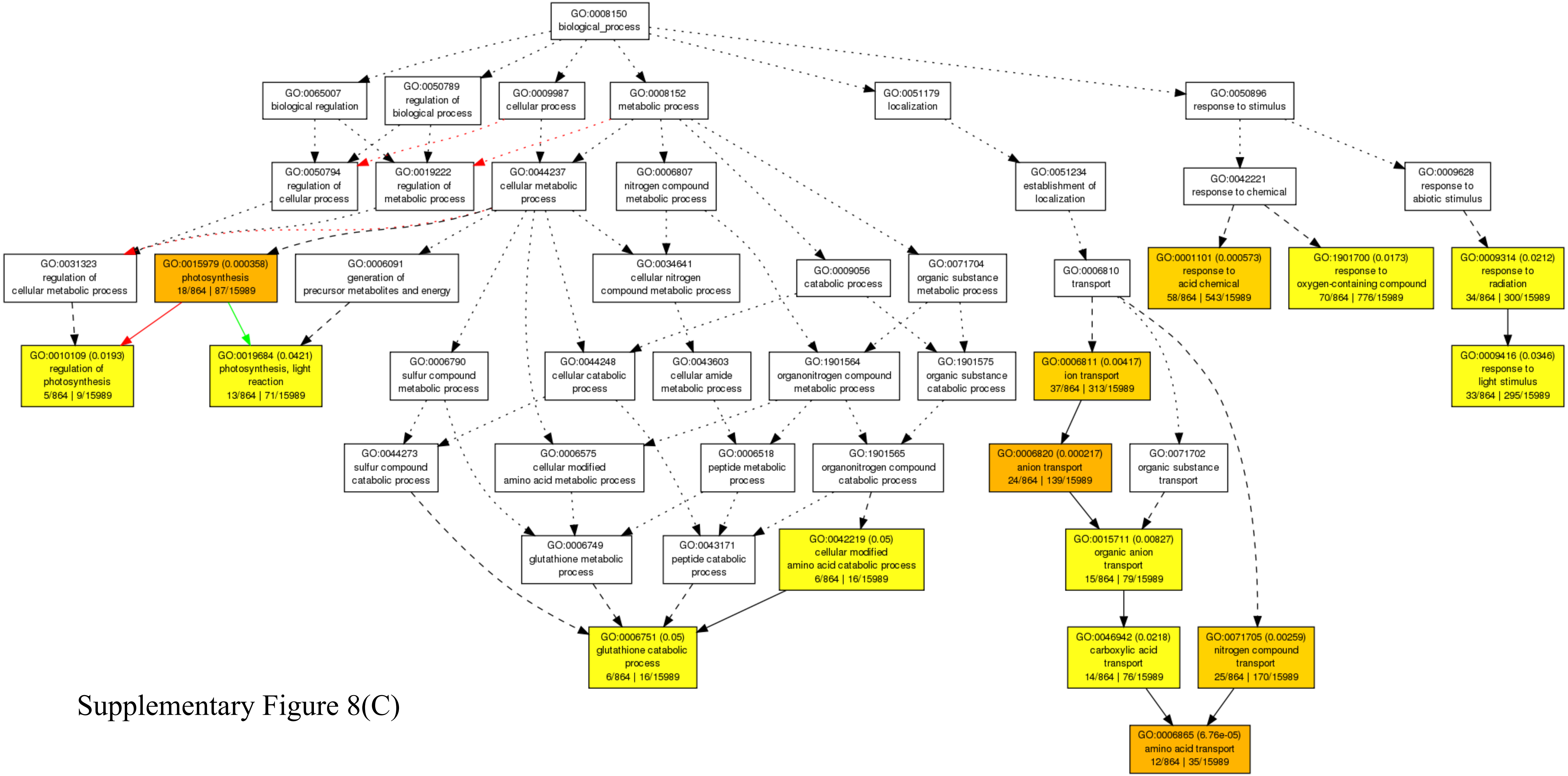

Supplementary Figure 8(C)

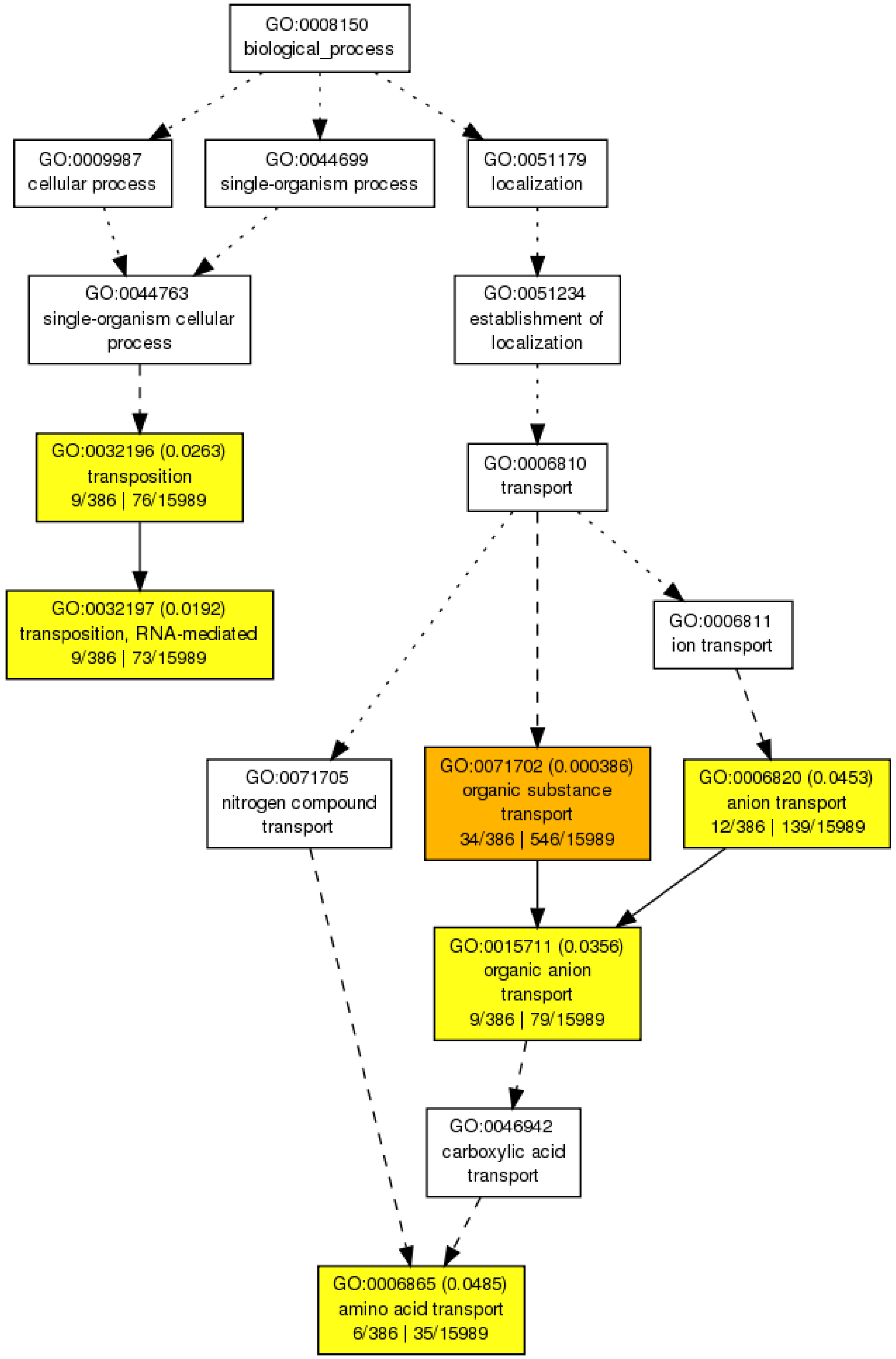

Supplementary Figure 8(D)

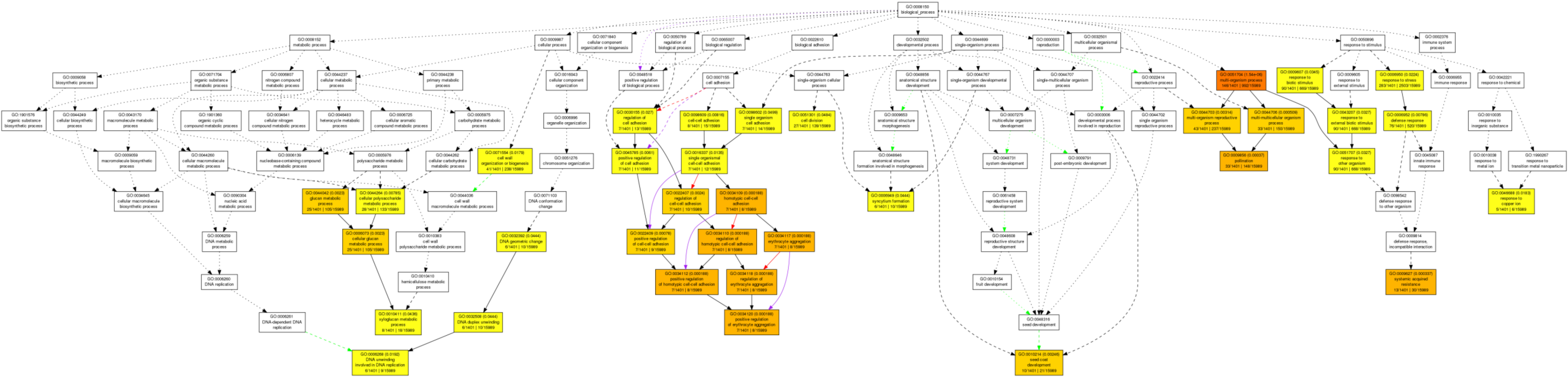

Supplementary Figure 8(E)

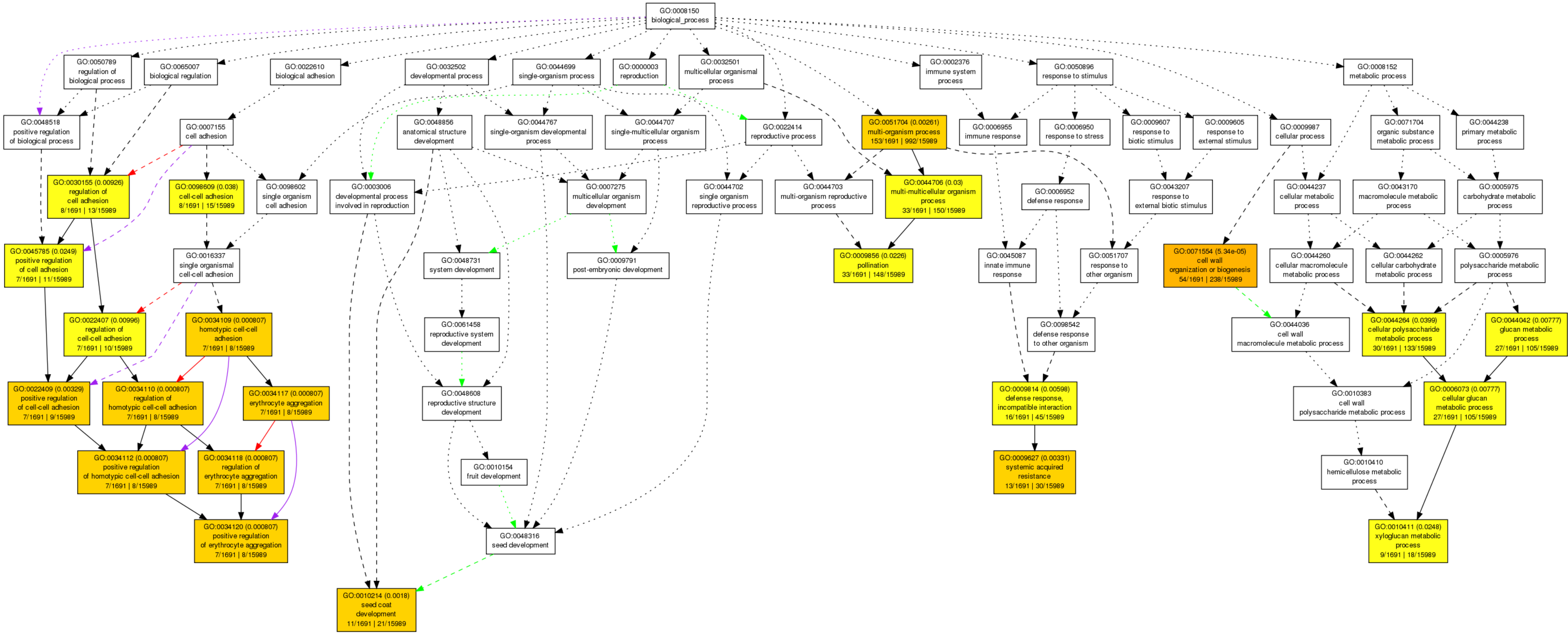

Supplementary Figure 8(F)

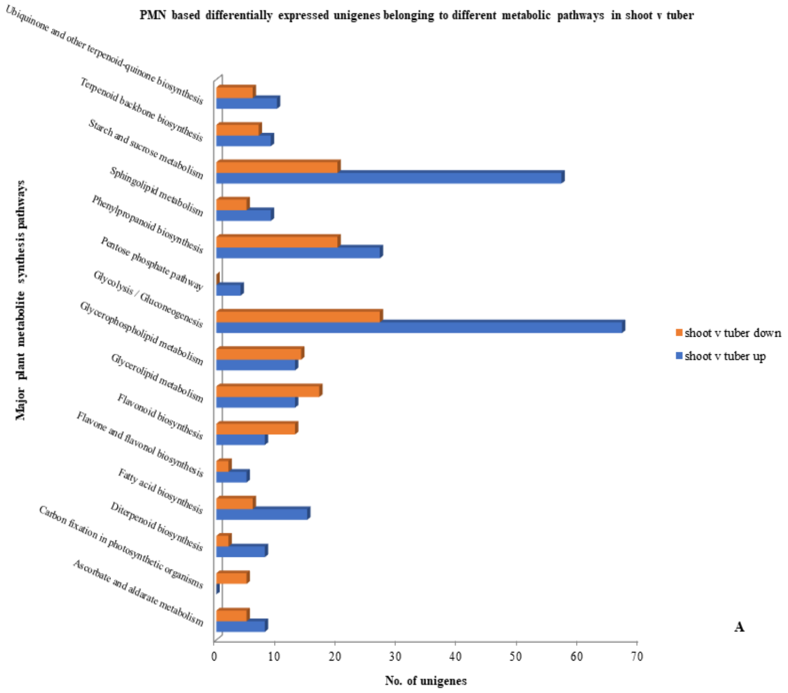

Figure S9(A):

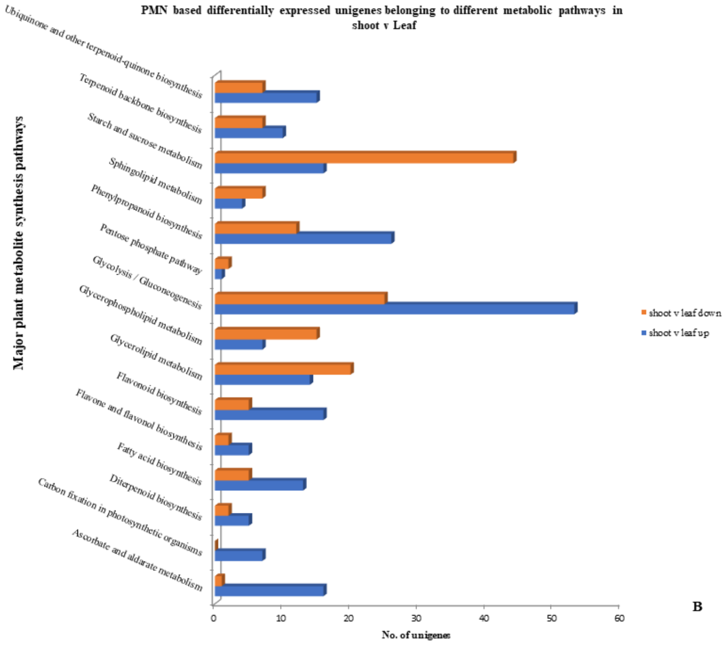

Figure S9(B):

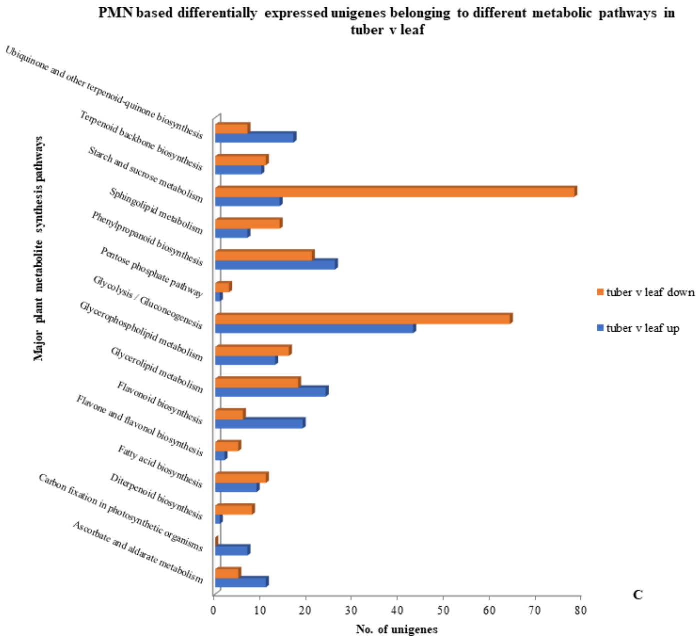

Figure S9(C):

Supplementary Figure 9: Distribution of the differentially expressed unigenes involved in different metabolite pathways based on PMN annotations (A) Shoot vs Tuber, (B) Shoot v Leaf, (C) Tuber v Leaf

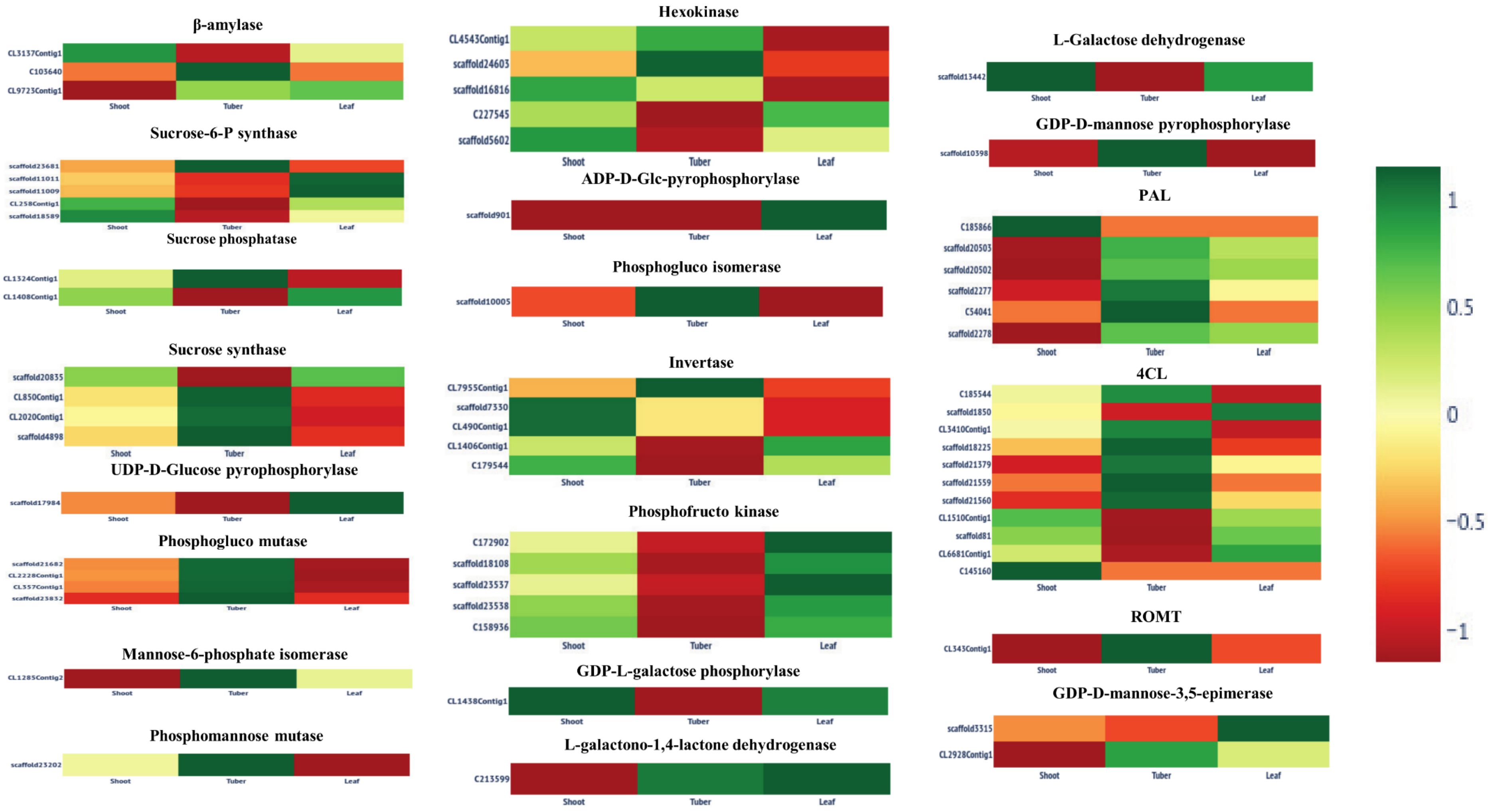

Supplementary Figure S10: Heatmaps of differentially expressed unigenes involved in carbohydrate partitioning and secondary metabolite biosynthesis in *D. hatagirea*

Supplementary Tables:

**Supplementary Table S1: Summary of sequencing statistics showing reads obtained for each plant part**						
**Plant part/tissue**	**Initial reads * 2**		**Final reads * 2**		**Final reads (%)**	
	**Rep 1**	**Rep 2**	**Rep 1**	**Rep 2**	**Rep 1**	**Rep 2**
**Shoot**	2,0540,112	2,19,87,864	1,47,84,544	1,61,28,304	71.98	73.35
**Tuber**	2,52,87,668	2,64,80,796	1,71,98,240	1,83,78,772	68.01	69.40
**Leaf**	2,06,14,976	2,32,04,504	1,49,06,468	1,69,28,080	72.31	72.95

**Supplementary Table S2: Details of the transcriptome data assembly statistics at different kmer in *D. hatagirea***						
**Kmer**	**Total Sequences**	**Maximum Length**	**Transcripts above 1000 bp (%)**	**N50**	**Total Basepair**	**Average Length**
21	88578	11896	13.386	1032	40342376	455.445
23	93741	11892	13.071	1016	41947153	447.479
25	98821	11892	12.763	1005	43059000	435.727
27	104594	11892	12.400	994	44464029	425.111
29	110361	11892	11.754	959	45107672	408.728
31	115150	11892	11.877	989	47166466	409.609
33	126304	11811	10.933	942	48904655	387.198
35	122024	11813	11.363	945	48677861	398.920
37	114962	11820	12.167	974	47970444	417.272
39	108528	11820	12.699	980	46992273	432.997
41	101262	11820	13.308	986	45611092	450.427
43	90425	11830	14.915	1034	43862311	485.068
45	81907	11829	15.977	1046	41794613	510.269
47	74139	10928	16.743	1048	39213252	528.915
**49**	**65384**	**10934**	**17.786**	**1057**	**36033299**	**551.103**
51	65733	10956	16.283	1027	33556917	510.503
53	57493	7759	16.322	1007	29543759	513.867
55	48801	8106	15.936	972	24966535	511.599
57	45889	7759	9.174	675	18986850	413.756
59	35088	7740	8.815	657	14564542	415.086

61	24504	7740	12.084	754	12058907	492.120
63	16702	7635	12.040	747	8494672	508.602
65	10384	5515	11.383	709	5291028	509.537
67	4998	4932	10.924	679	2535059	507.215

Supplementary Table S3: Summary of the transcriptome data, analysed using different programs

**Program used**	**Total sequences**	**Maximum length**	**Transcripts above 1000 bp (%)**	**N50**	**Total base pair**	**Average length**
SoapDenovo	65384	10934	17.79	1057	36033299	551.10
TGICL-CAP3	52581	10934	21.95	1121	33889035	644.51
CD-HIT	43873	10934	19.09	1029	26418584	602.16
DS Clustering	35371	10934	19.060	977	22994105	650.083

Legends of Supplementary Figures and Tables:

Supplementary Figures:

Supplementary Figure S1: Summary of sequencing statistics showing reads obtained for each plant part

Supplementary Figure S2: RPS blast against CDD database showing RRM_SF and Pkc_like maximally enriched term.

Supplementary Figure S3: Pooled datasets showing distribution of unigenes in *D. hatagirea* across GO classification (a) biological, (b) molecular and (c) cellular process categories; where (i-iii) represent differential expression in (i) shoot-versus-tuber, (ii) shoot-versus-leaf (iii) tuber- versus-leaf

Supplementary Figure S4: Pooled datasets showing distribution of unigenes in *D. hatagirea* across KEGG pathways where (i-iii) represent differential expression in (i) shoot-versus-tuber, (ii) shoot-versus-leaf (iii) tuber- versus-leaf

Supplementary Figure S5: Pooled dataset showing the distribution of unigenes in *D. hatagirea* across various categories of enzyme classification pathways where (i-iii) differential expression in (i) shoot-versus-tuber, (ii) shoot-versus-leaf (iii) tuber-versus-leaf

Supplementary Figure S6: Distribution of unigenes in *D. hatagirea* across various species on the basis of blast similarity search

Supplementary Figure S7: Bar graph showing total number of DEG's (log fold change ≥ 2) in different plant parts (a) down-regulated (b) up-regulated

Supplementary Figure S8: GO enrichment analysis of different plant parts showing enriched GO categories in (A) leaves in comparison to shoot, (B) leaves in comparison to tuber, (C) shoot in comparison to tuber, (D) shoot in comparison to leaves, (E) tuber in comparison to shoot, (F) tuber in comparison to leaf

Supplementary Figure S9: Distribution of the differentially expressed unigenes involved in different metabolite pathways based on PMN annotations (A) Shoot vs Tuber, (B) Shoot v Leaf, (C) Tuber v Leaf

Supplementary Figure S10: Heatmaps of differentially expressed unigenes involved in carbohydrate partitioning and secondary metabolite biosynthesis in *D. hatagirea*

Supplementary Tables:

Supplementary Table S1: Summary of sequencing statistics showing reads obtained for each plant part

Supplementary Table S2. Details of the transcriptome data assembly statistics at different kmer in *D. hatagirea*

Supplementary Table S3: Summary of the transcriptome data of *D. hatagirea*, analysed using different programs

Supplementary Table S4: List of DEG's above log fold change ≥ 4

Supplementary Table S5: Details of the selected differential expressing unigenes involved in various steps of plant response to cold stress in *D. hatagirea*

Supplementary Table S6: KEGG annotations of genes of leaves, shoot and tuber of *D. hatagirea* enriched under GO enrichment categories

Supplementary Table S7: PMN annotations for identification of unigenes involved in some important metabolite pathway

Supplementary Table S8: Transcription factor families identified in *D. hatagirea* transcriptome

Supplementary Table S9: List of contigs of differentially expressed unigenes involved in carbohydrate partitioning and secondary metabolite biosynthesis

Supplementary Table S10: Details of the differentially expressed transcripts involved in stress response and secondary metabolite biosynthesis in different tissue of *D. hatagirea*

Supplementary Table S11: Details of primers sequences of differentially expressing genes selected for qPCR validation
